# The yeast kinesin-5 Cin8 interacts with the microtubule in a noncanonical manner

**DOI:** 10.1074/jbc.M117.797662

**Published:** 2017-07-12

**Authors:** Kayla M. Bell, Hyo Keun Cha, Charles V. Sindelar, Jared C. Cochran

**Affiliations:** From the ‡ Department of Molecular and Cellular Biochemistry, Indiana University, Bloomington, Indiana 47405,; the § Department of Cell Biology, Yale School of Medicine, and; the ¶ Department of Molecular Biophysics and Biochemistry, Yale University, New Haven, Connecticut 06520

**Keywords:** ATPase, cryo-electron microscopy, enzyme mechanism, kinesin, kinetics, microtubule, molecular motor, Saccharomyces cerevisiae, thermodynamics, thermodynamics

## Abstract

Kinesin motors play central roles in establishing and maintaining the mitotic spindle during cell division. Unlike most other kinesins, Cin8, a kinesin-5 motor in *Saccharomyces cerevisiae,* can move bidirectionally along microtubules, switching directionality according to biochemical conditions, a behavior that remains largely unexplained. To this end, we used biochemical rate and equilibrium constant measurements as well as cryo-electron microscopy methodologies to investigate the microtubule interactions of the Cin8 motor domain. These experiments unexpectedly revealed that, whereas Cin8 ATPase kinetics fell within measured ranges for kinesins (especially kinesin-5 proteins), approximately four motors can bind each αβ-tubulin dimer within the microtubule lattice. This result contrasted with those observations on other known kinesins, which can bind only a single “canonical” site per tubulin dimer. Competition assays with human kinesin-5 (Eg5) only partially abrogated this behavior, indicating that Cin8 binds microtubules not only at the canonical site, but also one or more separate (“noncanonical”) sites. Moreover, we found that deleting the large, class-specific insert in the microtubule-binding loop 8 reverts Cin8 to one motor per αβ-tubulin in the microtubule. The novel microtubule-binding mode of Cin8 identified here provides a potential explanation for Cin8 clustering along microtubules and potentially may contribute to the mechanism for direction reversal.

## Introduction

Eukaryotic cells rely on cytoskeletal motor proteins for a variety of essential functions, including intracellular transport along microtubule tracks, mitotic spindle formation and maintenance for chromosome segregation during cell division, and cellular motility. Budding yeast *Saccharomyces cerevisiae* utilizes six cytoskeletal motors (five kinesins, Cin8, Kip1, Kip2, Kip3, and Kar3 and 1 cytoplasmic dynein, Dyn1) to produce motility, microtubule attachment, and regulation of microtubule dynamics in the mitotic spindle (1–3). Although there is a relatively limited number of spindle motors in yeast, none are essential for cell viability, suggesting these motors share functional overlap in their activities (1). Cin8 and Kip1 are members of the kinesin-5 (BimC) subfamily, which participate in microtubule cross-linking, antiparallel microtubule sliding, and microtubule destabilization for spindle assembly, spindle elongation, and chromosome congression in budding yeast (4–11).

Kinesin-5 motors from various eukaryotic organisms generally share structural similarity (especially within the ATPase motor domain). Four kinesin-5 heavy chains associate to form a bipolar homotetrameric quaternary structure with a pair of motor domains at each end of the tetramer long axis (12–14). High resolution structures of the human kinesin-5 (Eg5/KSP/KIF11) ATPase domain have been determined in different nucleotide states via X-ray crystallography (15, 16), cryo-electron tomography (17), and cryo-electron microscopy (cryo-EM) (18–20). The ATPase mechanism of truncated Eg5 motors has been characterized in numerous previous studies (21–34), which has demonstrated tight, stoichiometric (1:1) binding of the Eg5 motor domain with each αβ-tubulin dimer in the microtubule lattice. Eg5 utilizes coordinated ATPase cycles between its four motor heads to undergo slow, plus-end-directed movement on the microtubule (plus-stepping) (35, 36). However, it is not well understood how kinesin motors maintain this directional bias while stepping.

In mono-directional motors such as plus-stepping conventional kinesin (kinesin-1) or minus-stepping Ncd (kinesin-14), directionality has been attributed primarily to critical interactions between the neck/neck linker and the catalytic ATPase core (37–44). Chimeric motors containing the catalytic core from a minus-stepping motor (kinesin-14) and neck regions from a plus-stepping motor (kinesin-1) maintained plus-stepping (43, 45). Likewise, chimeric motors containing the catalytic core from a plus-stepping motor (kinesin-1) and neck regions from a minus-stepping motor (kinesin-14) maintained minus-stepping (37). Together, these studies suggest that directionality is determined by regions outside the catalytic ATPase core (44). However, several recent papers have demonstrated bidirectional motility by yeast kinesin-5 motors along microtubules (46–52), which has raised new questions about how directionality is determined in the kinesin superfamily.

Several kinesin-5 motors from different yeast species display bidirectional motility along microtubules (46–50, 53). Cin8 has been shown to move in both directions along the microtubule *in vitro* and *in vivo*, and it switches directionality based on ionic strength, relative microtubule orientation (*i.e.* parallel *versus* antiparallel interaction), and motor density (46–48). Additionally, bidirectional movement in a stable dimeric Cin8/kinesin-1 chimera (consisting of the Cin8 catalytic core and neck linker fused to the kinesin-1 stalk) has supported the hypothesis that Cin8 has intrinsic bidirectionality within the motor domain, with directional switching regulated by the C-terminal tails (52). Like Eg5, Cin8 shows predominantly slow plus-stepping when bound between antiparallel microtubules, and under low ionic strength, single Cin8 motors plus-step along individual microtubules (46, 47). However, at higher ionic strength, single Cin8 motors minus-step along the microtubule (47, 48). In surface gliding assays, increasing the Cin8 motor density results in directional switching from minus-stepping at low motor density to plus-stepping at high motor density (46). Loop 8 has been shown to influence directional switching such that when the large (99-residue) insert in loop 8 was removed, the Cin8 motor lost its ability to bind the microtubule at higher ionic strength, and the switch from minus-stepping at high ionic strength to plus-stepping at low ionic strength occurs at a much lower salt concentration (47).

Here, we have investigated microtubule interactions of the truncated Cin8 ATPase motor domain under different ionic strength conditions using thermodynamic, kinetic, and structural methodologies. We have discovered an unusual microtubule-binding behavior for Cin8 in direct comparison with the well-studied monomeric Eg5. We propose that these novel “noncanonical” binding interactions may be relevant to the mechanism for directional switching of Cin8 along the microtubule.

## Results

### Cin8 motor domains were pure, active, and monomeric in solution

All Cin8 motor domain constructs were 90–95% pure based on densitometry (supplemental Fig. S1) and demonstrated very slow basal activity with ∼20-fold enhancement of microtubule-stimulated ATPase activity at low ionic strength (supplemental Fig. S2*A*). This observed level of microtubule stimulation was comparable with Eg5 (∼70-fold stimulation under similar conditions). We utilized size-exclusion chromatography to establish the oligomeric state of our purified Cin8 protein (supplemental Fig. S2*B*). In comparison with monomeric Eg5, the Cin8 motor domain demonstrated comparable retention volumes, consistent with these motors existing as monomers in solution. There was no evidence of larger complexes of Cin8 in solution. Together, these Cin8 constructs provided advantages and disadvantages for various methodologies employed in this study (*e.g.* being able to resolve Cin8 from tubulin using SDS-PAGE), and through various biochemical experiments, they have given the general behavior of the Cin8 motor domain. These results provide evidence for active kinesins for in depth kinetic and thermodynamic characterization of the Cin8 motor domain.

### Steady-state kinetics reveal typical microtubule-stimulated kinesin ATPase activity

**Figure 1.**
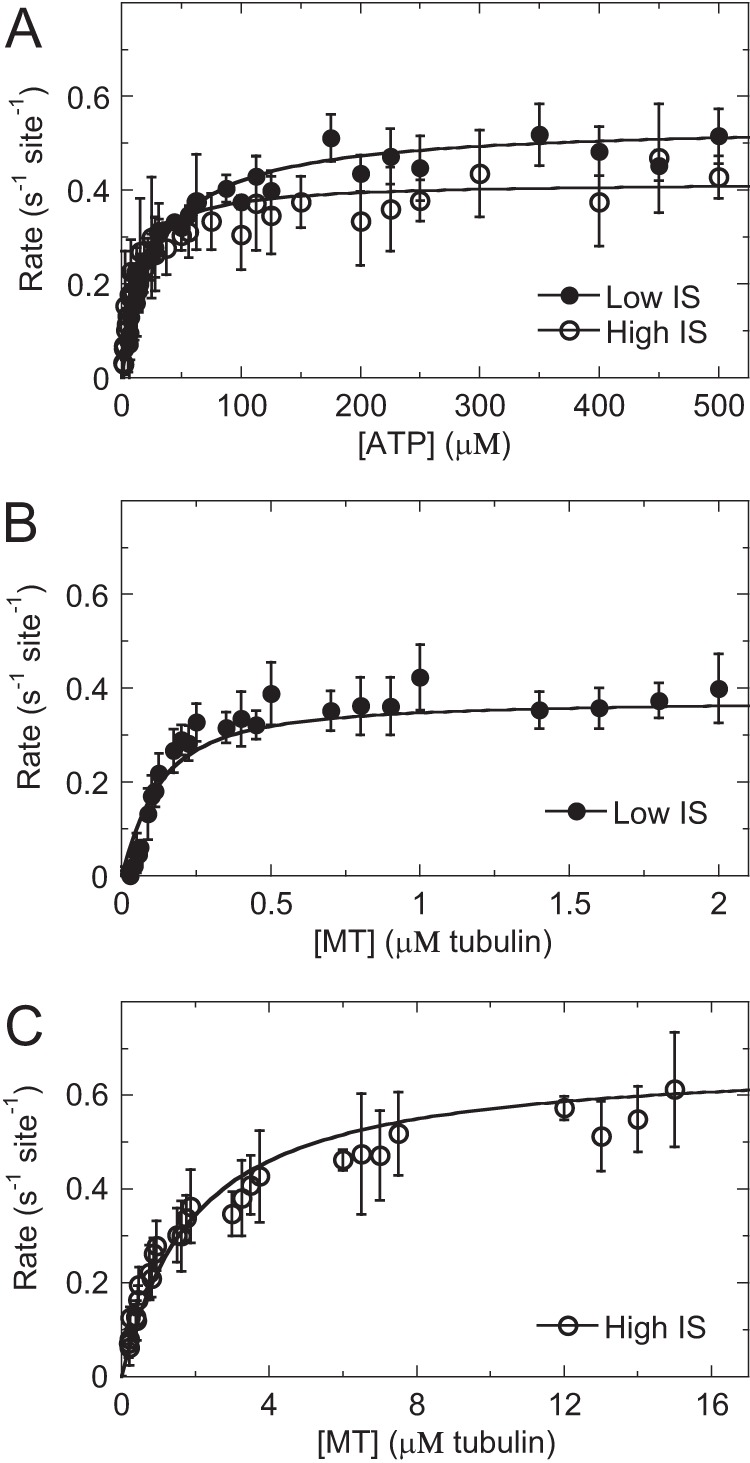
**Steady-state kinetics of Cin8 motors reveals typical microtubule-stimulated ATPase.** *A,* ATP-stimulated activity for Cin8 at low (*closed circles*) and high (*open circles*) ionic strength (*IS*). Final concentrations are 0.1 μm Cin8-His, 2 μm microtubules, and 5–1000 μm ATP. Data were fit to Equation 1. *Low, k*_cat_ = 0.54 ± 0.01 s^−1^ site^−1^ and *K_m_*_, ATP_ = 29 ± 2 μm. *High, k*_cat_ = 0.42 ± 0.01 s^−1^ site^−1^ and *K_m_*_, ATP_ = 12 ± 1 μm. *B,* microtubule-stimulated ATPase activity for Cin8 at low ionic strength. Final concentrations are 0.01 μm Cin8-His, 0–2 μm microtubules, and 1 mm ATP. Data were fit to Equation 2 to yield *k*_cat_ at 0.38 ± 0.01 s^−1^ site^−1^ and *K*_0.5, MT_ = 0.08 ± 0.02 μm. *C,* microtubule-stimulated activity for Cin8 at high ionic strength. Final concentrations are 0.2 μm Cin8, 0–30 μm microtubules, and 1 mm ATP. Data were fit to Equation 2 to yield *k*_cat_ at 0.68 ± 0.02 s^−1^ site^−1^ and *K*_0.5, MT_ = 1.8 ± 0.2 μm. *k*_basal_ was 0.01 ± 0.02 s^−1^ site^−1^ at low and 0.02 ± 0.02 s^−1^ site^−1^ at high ionic strength.

We performed a thorough steady-state kinetic analysis on all our Cin8 motor domains (supplemental Figs. S3 and S4 and supplemental Table S1). Fig. 1 shows the steady-state kinetics of the Cin8 motor domain as a function of ATP or microtubule concentration or ionic strength. Monomeric Cin8 motor domains showed relatively tight ATP binding that was comparable at low (*K_m_*_, ATP_ = 29 ± 16 μm) and high (*K_m_*_, ATP_ = 11 ± 2 μm) ionic strength (Table 1). Cin8 showed tight binding to microtubules at low ionic strength (*K*_0.5, MT_ = 0.08 ± 0.03 μm) that weakened ∼20-fold at high ionic strength (*K*_0.5, MT_ = 1.8 ± 0.3 μm). The Cin8 *k*_cat_ was ∼0.5 ± 0.1 s^−1^ site^−1^ under both low and high ionic strength, suggesting an independence of the rate-limiting constant on ionic strength. All Cin8 motor domain constructs demonstrated comparable steady-state kinetic constants (supplemental Table S1), suggesting the purification tags did not interfere with microtubule or ATP binding. The steady-state kinetics of the loop 8 deletion mutant of Cin8 (Cin8-His-Δ99) were also investigated at low and high ionic strength (supplemental Fig. S3, *C* and *D*). Its *k*_cat_ at 0.4 ± 0.2 s^−1^ site^−1^ was like the wild-type Cin8 motor domain with comparable ATP-binding affinity (*K_m_*_, ATP_ = 45 ± 4 μm) and ∼20-fold weaker microtubule binding (*K*_0.5, MT_ = 1.3 ± 0.9 μm) under low ionic strength (Table 1).

**Table 1:** **Steady state kinetic constants for kinesin-5 motors**

Ionic strength	Eg5, 21 mm	Cin8-His		Cin8-His-Δ99, 21 mm
		21 mm	88 mm	
*k*_cat_ (s^−1^ site^−1^)	2.5 ± 0.3*^a^*	0.38 ± 0.04	0.68 ± 0.13	0.36 ± 0.22
*K_m_*_, ATP_ (μm)	9.5 ± 0.4*^b^*	29 ± 16	11 ± 2	45 ± 4
*K*_0.5, MT_ (μm)	0.7 ± 0.6*^b^*	0.08 ± 0.03	1.8 ± 0.3	1.3 ± 0.9

*^a^* Eg5 constants were previously determined at 68.

*^b^* Average fit constants ± S.D. *n* = 3–6 experimental data sets for each condition.

### Cin8 motor domains show super-stoichiometric interaction with the microtubule lattice

**Figure 2.**
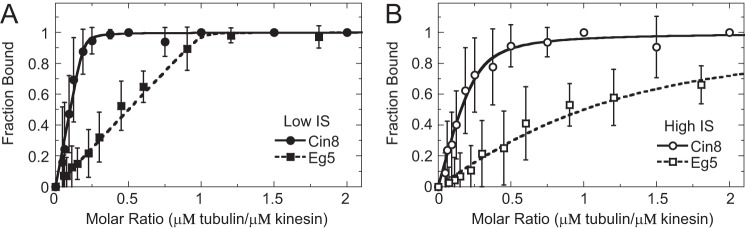
**Cosedimentation assays demonstrate super-stoichiometric Cin8 interaction with microtubules.** Cosedimentation assays were used to monitor Cin8 (*circles*) and Eg5 (*squares*) binding to microtubules at low (*closed symbols*) and high (*open symbols*) ionic strength (*IS*). *A,* Cin8-trx and Eg5 cosedimentation results at low ionic strength. Final concentrations are 2 μm kinesin and 0–4 μm microtubules. The data were fit to Equation 3 allowing *K_d_*_, app_ (both Cin8 and Eg5) and *s* (Cin8 only) constants to float in the analysis. Cin8, *K_d_*_, app_ = 0.013 ± 0.013 μm and *s* = 5.1 ± 0.3. Eg5, *K_d_*_, MT_ = 0.001 ± 0.004 μm. *B,* Cin8-trx and Eg5 cosedimentation results at high ionic strength. Final concentrations are 2 μm kinesin and 0–4 μm microtubules. Cin8, *K_d_*_, app_ = 0.13 ± 0.06 μm and *s* = 4.0 ± 0.5. Eg5, *K_d_*_, MT_ = 0.50 ± 0.06 μm.

The equilibrium binding affinity of the Cin8 motor domain for microtubules in the absence of added nucleotide was measured using cosedimentation assays. At low ionic strength, Cin8 (2 μm) partitioned with the microtubules as a function of microtubule concentration with 99.9% maximal binding at ∼0.5 μm paclitaxel-stabilized microtubules (Fig. 2*A*). These results indicated that 4–6 Cin8 motor domains bound tightly per αβ-tubulin heterodimer along the microtubule lattice (*K_d_*_, app_ ≈1 nm; Table 2). At high ionic strength, ∼3–5 Cin8 motor domains bound per tubulin dimer with ∼130-fold weaker binding affinity (*K_d_*_, app_ ≈130 nm; Table 2). To confirm that this observed super-stoichiometric binding of Cin8 to microtubules was not due to the error in protein concentration determination, Cin8 and Eg5 motor domains were mixed and cosedimented using identical aliquots from a single microtubule serial dilution. At both low and high ionic strength, Eg5 bound microtubules in the expected 1:1 ratio with very tight binding affinity at low ionic strength (*K_d_* ≈14 nm) and ∼35-fold weaker binding at high ionic strength (*K_d_* ≈500 nm). Super-stoichiometric binding to microtubules was observed for all Cin8 constructs (supplemental Fig. S5 and Table 2), suggesting that Cin8 interacts with the microtubule lattice such that 3–5 motor domains were bound per tubulin dimer with relatively tight binding affinity (supplemental Fig. S6).

**Table 2:** **Microtubule equilibrium binding constants for kinesin-5**

Ionic strength	Eg5		Cin8-Trx		Cin8-His		Cin8-His (ATP)	
	21 mm	88 mm	21 mm	88 mm	21 mm	88 mm	21 mm	88 mm
*K_d_*_,__app_ (μm)*^a^*	0.014 ± 0.007	0.50 ± 0.22	0.001 ± 0.003	0.13 ± 0.39	0.26 ± 0.15	0.18 ± 0.12	0.14 ± 0.06	0.96 ± 0.45
CI_MSE_ (μm)*^b^*	0.005–0.029	0.36–5.37	<1 × 10^−6^–0.04	0.06–0.25	0.01–0.63	0.01–0.23	0.02–0.62	0.44 to>1000
*s**^c^*	1.1 ± 0.7	1.4 ± 0.7	5.1 ± 4.2	4.0 ± 3.3	3.1 ± 0.6	3.0 ± 1.0	3.7 ± 2.1	6.1 ± 4.8
CI_MSE_	1.07–1.19	0.85–5.85	4.68–5.71	3.35–4.95	2.66–7.85	2.42–4.19	2.66–7.85	2.58 to >100

*^a^* Average fit constants when stoichiometry coefficient (*s*) held fixed to 1 for Eg5 and floated for Cin8 ± standard deviation of the mean is shown. *n* = 3–6 experimental data sets for each condition.

*^b^* CI_MSE_ is the asymmetric confidence interval determined from our MSE analysis.

*^c^* Stoichiometry coefficient (*s*) was determined by floating both *K_d_*_,__app_ and *s* in Equation 3 using nonlinear least squares fitting in Mathematica.

**Figure 3.**
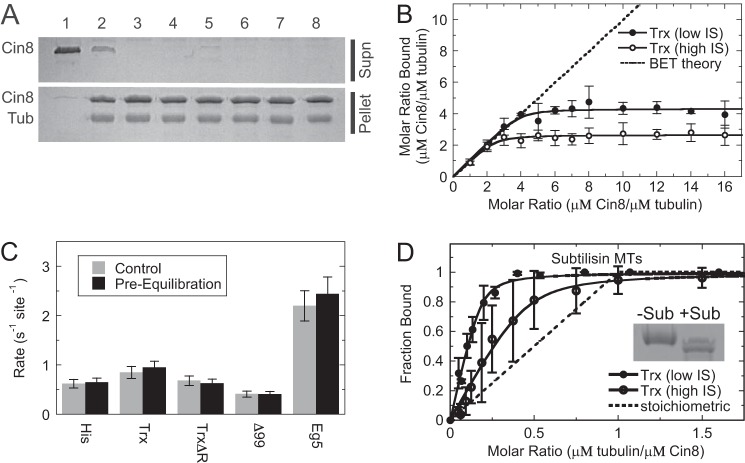
**Control reactions show defined, reversible super-stoichiometric Cin8 binding to normal and subtilisin-treated microtubules.** *A,* cosedimentation results for Cin8-trx binding microtubules under various conditions. Final concentrations are 4 μm Cin8, 1 μm microtubules, and 20 μm paclitaxel. Cin8 only, *lane 1*; ATPase buffer, *lane 2*; ATPase buffer without glycerol, *lane 3*; ATPase buffer plus DTT, *lane 4*; 20 mm PIPES buffer, pH 6.9, *lane 5*; 20 mm Na_2_HPO_4_/NaH_2_PO_4_ buffer, pH 7.2, *lane 6*; 10 mm NaCl, *lane 7*; and 10 mm potassium acetate, *lane 8. B,* cosedimentation results at increasing super-stoichiometric ratios of Cin8-trx to a fixed amount of microtubules at low (*closed circles*) and high (*open circles*) ionic strength (*IS*). Final concentrations are 0.25–4 μm Cin8, 0.25 μm microtubules, and 20 μm paclitaxel. *Dashed line* represents linear model based on the BET adsorption theory. *C,* ATPase activity of kinesins after pre-equilibration of the motor with microtubules at a super-stoichiometric ratio (12:1 kinesin/tubulin). Control reactions correspond to reactions without pre-equilibration of the motor with microtubules. Final concentrations are 0.6 μm kinesin, 6 μm microtubules, and 1 mm ATP, NADH mixture. *D,* Cin8-trx cosedimentation results at low and high ionic strength conditions using subtilisin-treated microtubules, which lack the tubulin C-terminal tails. Final concentrations are 2 μm Cin8, 0–4 μm subtilisin-treated microtubules, and 20 μm paclitaxel. Low ionic strength: *K_d_*_, app_ = 0.07 ± 0.03 μm, *s* = 5.4 ± 0.4. High ionic strength: *K_d_*_, app_ = 0.08 ± 0.04 μm, *s* = 2.2 ± 0.2. *Inset* shows 10% SDS-polyacrylamide gel image for normal (−*Sub*) and subtilisin-treated (+*Sub*) microtubules.

To test whether our biochemical conditions were promoting the atypical microtubule-binding behavior of Cin8, cosedimentation assays were performed under a variety of conditions. Cin8 binding to microtubules was tested after the following: 1) removing glycerol from the ATPase buffer; 2) adding 2 mm dithiothreitol to the ATPase buffer to prevent nonspecific disulfide bond formation; 3) switching the buffer from HEPES to PIPES; 4) switching the buffer from HEPES to sodium phosphate; 5) switching the salt from potassium chloride to sodium chloride; and 6) switching the salt from potassium chloride to potassium acetate. Under all tested conditions, super-stoichiometric binding was observed with at least four motors bound per tubulin dimer (Fig. 3*A*). A cosedimentation experiment was designed at increasing molar ratios of Cin8/microtubule in the absence of added nucleotide. We observed hyperbolic saturation of the bound Cin8/microtubule molar ratio (Fig. 3*B*), suggesting a defined Cin8 oligomer bound to the microtubule lattice.

To test whether the Cin8 oligomerization on microtubules was reversible, we designed an experiment where a high concentration of Cin8 motor domains (6 μm) was pre-equilibrated for 30 min at super-stoichiometric ratios with microtubules (12 Cin8 motor domains per tubulin dimer as microtubules). If Cin8 oligomerization was reversible, then we expected similar activity after diluting the complex into excess microtubules (1 Cin8 motor domain per 10 tubulin dimers as microtubules) compared with control reactions performed side-by-side without the pre-equilibration step. If Cin8 oligomerization was irreversible, then we expected a decrease in activity by 33–50% based on our cosedimentation results at low ionic strength (Fig. 2*A*) where 4–6 Cin8 motors were complexed per tubulin dimer under similar super-stoichiometric conditions. We observed similar ATPase activity for all Cin8 motors (Fig. 3*C*) suggesting Cin8 oligomerization on the microtubule under super-stoichiometric conditions was reversible.

To test whether the C-terminal tails of tubulin were necessary for super-stoichiometric Cin8 binding to microtubules, cosedimentation experiments were designed at increasing molar ratios of Cin8/microtubule in the absence of added nucleotide at low and high ionic strength with microtubules that were treated with subtilisin to remove the C-terminal tails. Under both conditions, super-stoichiometric Cin8 binding was observed with stoichiometry coefficient (*s*) values of 5.4 ± 0.4 and 2.2 ± 0.2 at low and high ionic strength, respectively (Fig. 3*D*). These results indicate that the tubulin C-terminal tails are not required for promoting the oligomerization of Cin8 along the microtubule lattice.

### Competition between kinesin-5 binding to microtubules reveals Cin8 binding to both canonical and noncanonical microtubule-binding sites

**Figure 4.**
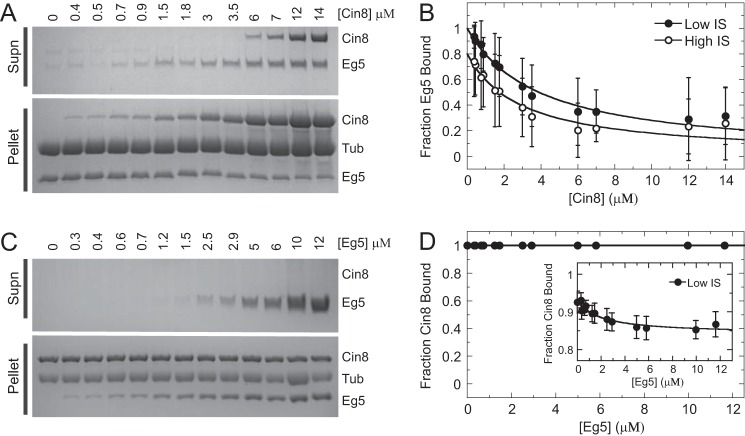
**Competition cosedimentation experiments reveal Cin8 binding to canonical and noncanonical microtubule-binding sites.** *A,* representative gel image of increasing Cin8 concentration at fixed microtubule·Eg5 concentration at high ionic strength (*IS*). Final concentrations are 2 μm Eg5, 2 μm microtubules, and 0–14 μm Cin8-trx. *B,* plot of average fraction Eg5 bound at increasing Cin8 concentration at low (*closed symbols*) and high (*open symbols*) ionic strength. Data were fit to Equation 5 to obtain *K_d_*_, app_ for Cin8 at low ionic strength (0.002 ± 0.0001 μm) and high ionic strength (0.59 ± 0.06 μm). *C,* representative gel image of increasing Eg5 concentration at fixed microtubule·Cin8 concentration at low ionic strength. Final concentrations are 2 μm Cin8-trx, 2 μm microtubules, and 0–12 μm Eg5. *D,* plot of average fraction Cin8 bound to microtubules at increasing Eg5 concentration (*circles*) when Cin8·microtubule was 1:1 as in *C.* The *inset* shows the fraction of Cin8 bound at increasing Eg5 when Cin8·microtubule was 4:1. *Supn,* supernatant.

Competition assays were performed between Cin8 and Eg5 for binding to microtubules to define how Cin8 oligomerizes when bound to the microtubule lattice. Monomeric Eg5 has been shown to bind tightly to the microtubule with a 1:1 stoichiometry at the “canonical” kinesin–microtubule-binding site (Fig. 2 and supplemental Fig. S7) (18–21, 54). If the Cin8 oligomer binds only to the canonical kinesin–microtubule-binding site and has a similar tight microtubule-binding affinity for this canonical site compared with monomeric Eg5 (Fig. 2), then increasing Cin8 concentrations should compete Eg5 off the microtubule when starting with a fixed Eg5/microtubule ratio (1:1). Likewise, increasing Eg5 concentrations should compete Cin8 off the microtubule when starting with a fixed Cin8/microtubule ratio (1:1 or 4:1). To ensure that any differences in the equilibrium binding behavior were not due to drastic kinetic differences between Cin8 and Eg5 dissociation from the microtubule, the motors were incubated with the microtubules for 1 h before cosedimentation. Increasing Cin8 concentrations promoted a decrease in the fraction Eg5 bound (Fig. 4, *A* and *B*), suggesting that Cin8 does bind to the canonical kinesin–microtubule-binding site; however, there was no change in fraction Cin8 bound (1:1 Cin8/microtubule) at increasing Eg5 concentrations (Fig. 4, *C* and *D*). Even when the canonical kinesin–microtubule-binding site was saturated with Eg5, no Cin8 was observed in the supernatant, suggesting that Cin8 was bound to another microtubule-binding site (referred to as the “noncanonical” site) that Eg5 was unable to compete for binding. When a similar experiment was conducted with a super-stoichiometric ratio of Cin8 to microtubules (4:1), increasing Eg5 concentration was only able to displace ∼10% of the bound Cin8, consistent with the presence of a noncanonical microtubule-binding site for Cin8.

### Super-stoichiometric microtubule binding is independent of nucleotide state, yet dependent on the Cin8 loop 8 insert

**Figure 5.**
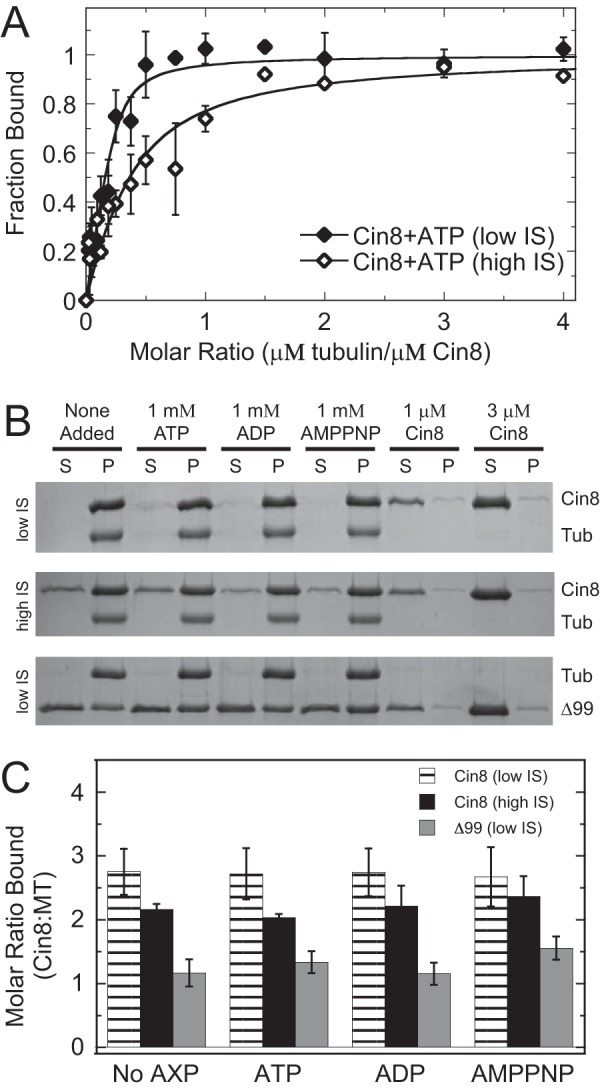
**Cin8 super-stoichiometric microtubule binding was independent of nucleotide state, yet dependent on the loop 8 insert.** *A,* results for Cin8 binding to microtubules in the presence of saturating ATP plus an ATP regeneration system at low (*closed diamonds*) and high (*open diamonds*) ionic strength (*IS*). Final conditions are 0.5 μm Cin8-His, 0–2 μm microtubules, 1 mm ATP, 2 mm phosphoenolpyruvate, 2 mm NADH, 5 units/ml pyruvate kinase, and 8 units/ml lactic dehydrogenase. The data were fit to Equation 1 allowing *K_d_*_, app_ and *s* constants to float in the analysis. Low: *K_d_*_, app_ = 0.11 ± 0.09 μm and *s* = 3.7 ± 0.8. High: *K_d_*_, app_ = 1.6 ± 1.6 μm and *s* = 6.1 ± 4.8. *B,* representative gel images showing supernatant (*S*) and pellet (*P*) samples for cosedimentation reactions using Cin8-trx and Cin8-His-Δ99 under low and high ionic strength with different excess nucleotide conditions (as indicated). Control reactions with 1 and 3 μm Cin8 only are shown. Final conditions are 3 μm Cin8, 1 μm microtubules, and 1 mm A*X*P (A*X*P refers to any adenosine nucleotide). *Tub*, tubulin. *C,* molar ratio bound to microtubules as a function of nucleotide state, Cin8 protein (Trx *versus* Δ99), and ionic strength as shown in *B. Error bars* report the standard deviation of the mean for each condition.

Cosedimentation experiments for Cin8 were performed in the presence of 1 mm ATP plus an ATP regeneration system, and the fraction bound was quantified using an NADH-coupled assay (Fig. 5*A*). The apparent microtubule-binding affinity for Cin8 under steady-state ATP turnover conditions was similar to its microtubule-binding affinity in the absence of added nucleotide (*K_d_*_, app_ = 0.14 ± 0.06 and 0.26 ± 0.15 μm, respectively) under low ionic strength. However, under high ionic strength, the apparent microtubule-binding affinity was ∼10-fold weaker for Cin8 under steady-state ATP turnover conditions compared with the absence of added nucleotide (*K_d_*_, app_ = 0.96 ± 0.45 and 0.18 ± 0.12 μm, respectively). Under both low and high ionic strength, Cin8 bound super-stoichiometrically to microtubules with stoichiometry coefficient (*s*) values at 3.7 ± 2.1 and 6.1 ± 4.8, respectively (Fig. 5*A*; Table 2).

Additionally, cosedimentation assays for Cin8 in a 3:1 ratio with microtubules under different excess nucleotide conditions (no added nucleotide, ATP, ADP, and AMPPNP^2^) showed super-stoichiometric binding at both low and high ionic strength (Fig. 5, *B* and *C*). However, there was no apparent difference in Cin8-binding affinity between nucleotide conditions under both low and high ionic strength. Removal of the large insert in loop 8 from the Cin8 motor domain (our Cin8-His-Δ99 construct) resulted in a dramatic shift to stoichiometric binding to microtubules (*s* = 1.3 ± 0.2) with no significant change in binding affinity at various nucleotide conditions (Fig. 5, *B* and *C*). These results suggest that loop 8 plays a critical role in promoting the super-stoichiometric binding observed by our three Cin8 motors likely through the promotion of Cin8·Cin8 oligomerization and binding to both canonical and noncanonical microtubule-binding sites.

### Presteady-state kinetics of kinesin-5-binding microtubules show rapid Cin8 interaction with the noncanonical microtubule site dependent on the loop 8 insert

**Figure 6.**
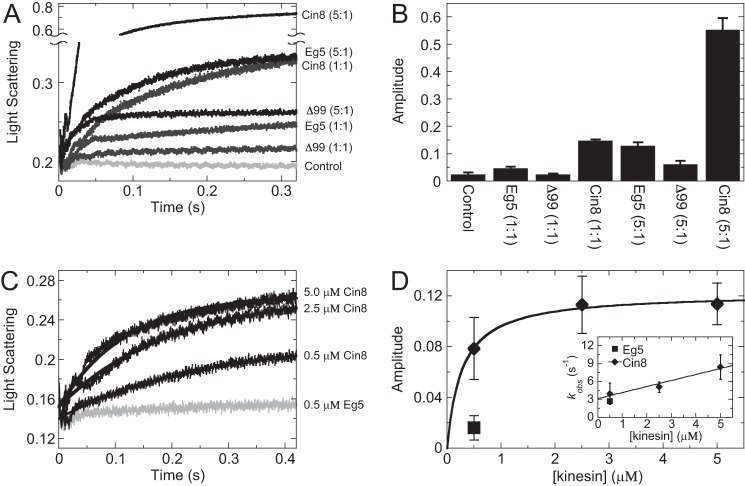
**Presteady-state kinetics of kinesin-5-binding microtubules show rapid Cin8 interaction with the noncanonical microtubule-binding site that was dependent on the loop 8 insert.** *A,* stopped-flow transients showing the change in light scattering as Cin8 or Eg5 were rapidly mixed with microtubules under stoichiometric (1:1) and super-stoichiometric (5:1) conditions at low ionic strength. Final concentrations are 0.5 or 2.5 μm Cin8-His, Cin8-His-Δ99 or Eg5, and 0.5 μm microtubules. Control reaction was light scattering from microtubules alone. *B, bar graph* showing the average transient amplitudes from *A* (*n* = 5–7 transients). *Error bars* denote the standard deviation of the mean. *C,* stopped-flow transients showing the change in light scattering as Cin8 was mixed with a pre-formed microtubule·Eg5 complex at low ionic strength. Final concentrations are 0.5–5 μm Cin8-His, 0.5 μm Eg5, and 0.5 μm microtubules. Control reaction (*gray*) corresponds to 0.5 μm Eg5 mixed with a pre-formed microtubule·Eg5 complex. *D,* plot showing the average transient amplitudes from *C* (*n* = 5–7 transients) for Cin8 or Eg5 as a function of kinesin concentration. Cin8 data were fit to Equation 6 to yield the *K*_0.5_ = 0.27 ± 0.05 μm. The *inset* shows a plot of the observed rate of binding that was fit to Equation 8: *K*_+1_*k*_+2_ = 1.0 ± 0.2 μm^−1^ s^−1^ and *k*_−2_ = 3.1 ± 0.7 s^−1^.

The presteady-state kinetics of kinesin binding to microtubules was measured via a change in light scattering upon Cin8 or Eg5 binding to microtubules in a stopped-flow instrument. When kinesin was rapidly mixed with microtubules at a stoichiometric ratio (1:1), comparable amplitudes were observed (Fig. 6, *A* and *B*, and supplemental Fig. S8). However, when kinesin and microtubules were mixed in a super-stoichiometric ratio (5:1 Cin8/microtubules), Cin8 binding to microtubules showed a significantly higher amplitude in light scattering compared with the Eg5 control, suggesting the average mass of the ensemble of microtubule·Cin8 complexes was higher than the average mass of the microtubule·Eg5 complexes. Performing similar presteady-state experiments with Cin8-His-Δ99, we observed sub-stoichiometric interaction with the microtubule lattice, even under super-stoichiometric conditions (Fig. 6, *A* and *B*, and supplemental Fig. S8). These results support the model for loop 8 being necessary for the super-stoichiometry of Cin8 binding to the microtubule lattice.

To test the hypothesis for a Cin8-specific noncanonical-binding site on the microtubule, Cin8 motor domain was rapidly mixed in a stopped-flow instrument with a preformed microtubule·Eg5 complex (Fig. 6*C*). There was a significant increase in amplitude suggesting that the Cin8 motor domain was binding rapidly to one or more noncanonical site(s) while Eg5 occupied the canonical site (Fig. 6*D*). The change in amplitude was plotted as a function of Cin8 concentration, which showed hyperbolic saturation, supporting a defined Cin8 oligomer bound to the microtubule lattice. The observed rate of Cin8 binding to microtubules increased linearly providing the apparent second-order rate constant for Cin8 association with microtubules at 1.1 ± 0.2 μm^−1^ s^−1^ and extrapolated the off-rate for Cin8 dissociation from the microtubule at 3.1 ± 0.2 s^−1^. These results indicated that, despite being noncanonical, the alternative binding site(s) has(have) comparable kinetic rate constants to other mitotic kinesin motors (21, 55, 56).

### High resolution structure of Cin8 bound to the canonical microtubule site reveals novel loop 8 interaction

**Figure 7.**
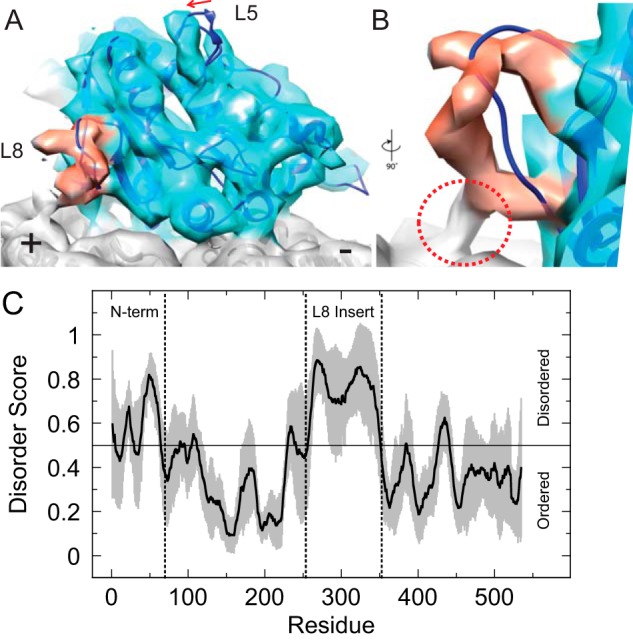
**Cryo-EM structure of the Cin8 motor domain bound to the canonical microtubule-binding site reveals novel loop 8 interaction with microtubule lattice.** *A,* three-dimensional reconstruction of Cin8-His (*light blue*) in the ADP·AlF*_x_* state bound to the microtubule (*gray*) at ∼7 Å resolution. The loop 8 density is highlighted in *red,* and the difference in loop 5 conformation compared with the Eg5 model is indicated with an *arrow*. The crystal structure of Eg5 bound to AMPPNP (*dark blue*, PDB code 3HQD) was placed into the density by rigid docking. *B,* close-up view of loop 8 rotated 90° and looking into the loop, which makes contact with the microtubule lattice (*red circle*). *C,* average score (*solid black line*) for disorder prediction for the Cin8 motor domain plus the N-terminal extension (residues 1–535) based on eight different algorithms (see “Experimental procedures”). *Gray zone* shows calculated standard deviation of the mean at each residue. *Vertical dotted lines* show boundaries for the N terminus and the loop 8 insert.

To gain a better structural understanding of how Cin8 interacts with the microtubule lattice, cryo-EM was utilized to generate a reconstruction of the Cin8 motor domain in the ADP·AlF*_x_* state bound to the canonical microtubule-binding site. For the sake of obtaining a high-resolution structure using helical reconstruction methods (57–59), we initially sought to generate samples with monomeric Cin8 bound to the microtubule lattice at a 1:1 binding stoichiometry. In agreement with previous studies (10), we have observed strong microtubule bundling activity by Cin8 at higher molar ratios. To circumvent this issue, Cin8 was limited to ∼0.9× molar ratio, and the microtubule·Cin8 complex was mixed immediately before cryogenic freezing without pelleting the complex, which could induce bundling. Although a small percentage of microtubules exhibited partial decoration, most of the observed microtubules showed tooth-like decoration, indicative of 1:1 binding (supplemental Fig. S9*A*). Our ∼7 Å resolution cryo-EM map of Cin8 very closely resembles the canonical microtubule binding configuration of well-studied kinesins such as kinesin-1 and Eg5 (Fig. 7*A* and supplemental Fig. S9*B*). Because a reliable atomic model of Cin8 is not yet available, the crystal structure of Eg5 bound to AMPPNP (PDB code 3HQD (16)) was docked into the density map using rigid body fitting. Although much of the conserved motor domain in the Eg5 model conforms to the Cin8 cryo-EM density map, two structural differences were observed. First, there was a moderate shift of loop L5 toward the microtubule plus-end in the Cin8 map when compared with the Eg5 model (Fig. 7*A*). Second, the loop 8 in the Cin8 map extends out further toward the plus-end of the microtubule compared with Eg5 and appears to contact the microtubule lattice (Fig. 7*B*). However, the extra density that would be expected for the 99-residue extension within loop 8 was missing in our reconstruction, and it remains unclear precisely where the insertion lies within loop 8; at least two protrusions from the main path of the loop were visible, which may be candidates for the beginning and/or end of the extension. These results were consistent with the predicted disorder in loop 8 using eight different algorithms (Fig. 7*C* and supplemental Fig. S10). Together, these structural results suggest Cin8 preferentially binds the canonical microtubule-binding site when Cin8 concentration was sub-stoichiometric to microtubule sites.

To further explore the binding behavior of Cin8, we investigated whether the super-stoichiometric assembly could be visualized using cryo-EM. First, the molar ratio of Cin8 compared with tubulin dimers was increased in the presence of ADP·AlF*_x_*. When >1.3× molar excess of Cin8 was added to microtubules at low ionic strength, strong microtubule-bundling activity by Cin8 was observed, even when Cin8·microtubule complexes were not pelleted (data not shown). Next, Cin8 binding behavior in the no-nucleotide state was investigated by adding apyrase at low ionic strength. Under these conditions, microtubule bundling was observed at even lower sub-stoichiometries, with microtubule bundling observed even with only ∼0.75× molar excess Cin8. Strikingly, the addition of apyrase and subsequent digestion of nucleotide induced the formation of “fuzzy” microtubules that appear to be decorated with Cin8 in a super-stoichiometric, but partially disordered, manner (supplemental Fig. S9, *C* and *D*). This activity was not dependent on the Cin8 molar ratio, because fuzzy filaments were observed with Cin8 molar ratios even as low as 0.5×. At this molar ratio, the amount of microtubule bundling was negligible, presumably due to most microtubules being completely bare. The degree of decoration was highly variable even within the same grid, with some filaments being more thickly decorated and others showing sparse, partial decoration. Super-stoichiometric decoration by Cin8 was highly cooperative, which left many microtubules completely bare, suggesting that the supply of Cin8 was entirely consumed by fuzzy filaments.

## Discussion

### Cin8 exhibits a novel microtubule-binding mode

We utilized a combination of classic cosedimentation and novel competitive cosedimentation assays to investigate the interaction of Cin8 with microtubules under low and high ionic strength. These assays were controlled using the well-studied motor domain construct of human kinesin-5 Eg5 (residues 1–368) as the standard for canonical kinesin–microtubule interaction. For all four wild-type Cin8 constructs, we observed super-stoichiometric microtubule binding with ∼3–5 Cin8 motors binding per tubulin dimer in the microtubule lattice. Using mean square error (MSE) analysis, this stoichiometry coefficient (*s*) was well constrained by the data (typically ±10–25%; supplemental Fig. S6; Table 2), indicating a reproducible, defined oligomerization state of Cin8 on the microtubule lattice that varies within experimental error estimates. Control experiments using identical microtubule stocks yielded expected stoichiometric microtubule binding for Eg5 (Fig. 2). Manipulating a wide variety of biochemical conditions (buffer, salt, and reducing agent, etc.) did not eliminate super-stoichiometric microtubule binding by Cin8 (Fig. 3). Additionally, super-stoichiometric Cin8 binding to microtubules persisted under steady-state ATP turnover conditions, suggesting this behavior is linked to Cin8's ATPase cycle. The tubulin C-terminal tails were not necessary for achieving the super-stoichiometric binding state for Cin8 (Fig. 3*D*).

**Figure 8.**
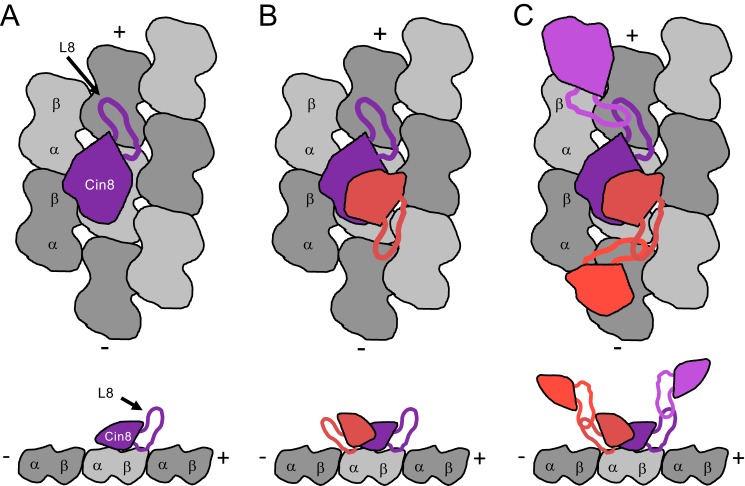
**Model for noncanonical microtubule interaction by Cin8 motors.** *A,* Cin8 on the canonical site is shown in *purple*. Tubulin dimers shown in *gray* make up the microtubule lattice. Top down view is shown at the *top,* and side view shown at the *bottom. B,* monomers of Cin8 on the canonical and noncanonical sites are shown in *purple* and *salmon*, respectively. The *salmon* Cin8 head is smaller than the *purple* Cin8 head to denote an alternative binding orientation for the Cin8 bound to the noncanonical microtubule-binding site via loop L8. *C,* dimers of Cin8 on the canonical microtubule-binding site are shown as *purple* and *magenta* and on the noncanonical binding site as *salmon* and *red*.

Given that Eg5 could not compete with the entire population of Cin8 bound to microtubules, these results strongly suggest that the Cin8 motor domain binds to one or more noncanonical-binding sites on the microtubule. With ∼3–5 Cin8 motors bound per tubulin dimer, a reasonable explanation for our data is that the microtubule binding somehow triggers Cin8 to oligomerize, thus explaining why Eg5 can compete off a fraction of the Cin8, at the same time as 3–5 Cin8 motors are observed to bind per tubulin dimer (Fig. 8). Our data cannot definitively establish the oligomeric state at both the canonical and noncanonical sites. The apparent binding affinity (*K_d_*_, app_) determined from fitting cosedimentation experimental data represents the average binding affinity for both canonical- and noncanonical-binding sites on the microtubule lattice as well as Cin8·Cin8 oligomerization. MSE analysis was employed to define asymmetric confidence intervals for *K_d_*_, app_ (supplemental Fig. S6), which was typically sub-micromolar or a low micromolar range, indicating an average tight binding affinity for all sites (Table 2). If this apparent tight interaction was due to irreversible denaturation and aggregation of the Cin8 motors, then we would expect two observations as follows: 1) if we increased the Cin8 motor concentration to higher super-stoichiometric ratios, we would see more and more Cin8 aggregate and pellet with the microtubules; and 2) if we pre-equilibrated the Cin8 motors with microtubules at a super-stoichiometric ratio, there would be decreased activity for the irreversibly aggregated motors in this complex. We did not observe either result (Fig. 3, *B* and *C*, respectively), suggesting this behavior is reversible and intrinsic to the Cin8 motor domain. Together these results support a model whereby Cin8 binds two independent sites on the microtubule and likely oligomerizes at one or both sites due to interactions via loop 8 (see below) (Fig. 8).

An apparent inconsistency exists when interpreting both competition assay results and the data from cryo-EM experiments. Cin8 motor domain appears to bind the canonical microtubule-binding site, yet under certain conditions it can bind at one or more noncanonical sites. We are quite confident from our competition assays (Fig. 4, *A* and *B*) as well as our cryo-EM experiments (Fig. 7) that the Cin8 motor domain can bind to the canonical microtubule-binding site. Our data from competition assays (Fig. 4, *C* and *D*), our presteady-state light-scattering experiments (Fig. 6, *C* and *D*), and our cryo-EM experiments (Fig. 7) at increasing Cin8/microtubule ratios (∼4:1) suggest that the Cin8 motor domain can bind to one or more noncanonical microtubule-binding sites, which Eg5 cannot compete for binding. One key difference between these experiments was the nucleotide conditions (no added nucleotide for competition experiments and ADP-AlF*_x_* for cryo-EM), which potentially could explain the difference in binding site preference. This question is somewhat difficult to resolve (at least structurally) because the nucleotide-free state has been heterogeneous to image by cryo-EM, perhaps indicating that Cin8 binds the noncanonical site first under these conditions (which would explain why Eg5 is less able to compete away the Cin8). Fig. 4*B* provides the most convincing evidence for comparable binding affinity between Eg5 and Cin8 for the canonical microtubule-binding site. Under low ionic strength, the apparent *K_d_* value for binding the canonical microtubule site was 0.001 and 0.002 μm for Eg5 and Cin8, respectively. Under high ionic strength, the *K_d_* was 0.50 and 0.59 μm for Eg5 and Cin8, respectively. Given the likely oligomerization of Cin8 on the microtubule, these binding constants are difficult to deconvolute but still set upper limits for comparison.

### Loop 8 insert is responsible for super-stoichiometric binding behavior in the Cin8 motor domain

The most prominent difference in the Cin8 kinesin motor domain is the 99-residue insert in loop 8 (residues 254–353). To investigate the role of this loop 8 insert in the microtubule-binding behavior of Cin8 motors, we purified a construct in which this 99-residue segment was substituted with the 7-residue loop 8 in the related *S. cerevisiae* kinesin-5 Kip1 as was done previously (14, 47). Interestingly, Cin8-His-Δ99 demonstrated near stoichiometric microtubule binding under all nucleotide conditions (Fig. 5, *B* and *C*), suggesting the loop 8 insert was somehow responsible for promoting the super-stoichiometric microtubule-binding behavior of the wild-type motor. This observation is novel and, to our knowledge, has not been seen in other kinesin motor domains studied to date. Nevertheless, the full-length Cin8-Δ99 protein shows bidirectional motility, which indicates that the mechanism for bidirectional motility is not solely linked to the super-stoichiometric microtubule-binding behavior. Loop 8 shows variability across the kinesin superfamily and has been implicated in microtubule-binding behavior of other kinesin motors (60–63). Our cryo-EM structure of the microtubule·Cin8 complex indicates that most of the unique loop 8 insertion is disordered, at least when Cin8 binds to the microtubule in the canonical mode (Fig. 7). Given that intrinsically disordered proteins can achieve different modes of macromolecular interaction, we hypothesize the disordered loop 8 when Cin8 is bound to the microtubule lattice promotes oligomerization of the Cin8 motor (Fig. 8), thus contributing to the super-stoichiometric microtubule binding.

### Potential functional roles for multiple Cin8 binding sites per tubulin dimer

Cin8 and other yeast kinesin-5 motors have been shown to possess bidirectional motility along microtubules (46–52). The hypothesized noncanonical microtubule-binding site along the lattice of the microtubule could lead to geometric constraints for getting Cin8 bound to a particular microtubule-binding site, which could provide an explanation for how Cin8 motor domains respond to both ionic strength and motor density along the microtubule lattice. Given the stoichiometry coefficient decreased with increasing ionic strength, we expect ionic strength to modulate the affinity between the canonical and noncanonical sites, which may contribute to the mechanism of reversal in directionality and clustering along the microtubule. One possible model to explain our biochemical and structural results as presented in Fig. 8 implies Cin8 oligomerization (likely dimerization) via loop 8 at both the canonical and noncanonical sites. The amino acid content of the large loop 8 insert in Cin8 includes short stretches of polyasparagine and polyglutamine, which have been shown to promote protein aggregation. Our data are consistent with the loop 8 insert facilitating higher order oligomerization of Cin8 upon binding the microtubule lattice. As indicated in our model (Fig. 8*C*), perhaps the differential orientation of Cin8 between canonical and noncanonical sites coupled to loop 8-dependent dimerization could bias the motor either toward the microtubule plus-end *versus* minus-end. Perhaps the recent studies by Shapira and Gheber (65) as well as Goldstein *et al.* (64), which showed loop 8-dependent post-translational modifications, would provide a means to regulate Cin8 activity *in vivo* via manipulation of the canonical/noncanonical or Cin8/Cin8 interfaces.

Recent studies have indicated Cin8 motor coupling via “cluster” formation whereby multiple tetrameric motors interact through noncovalent interactions (66). This cluster formation has been proposed to modulate the motility properties of Cin8, and the properties are likely important during yeast cell division for 1) the establishment of the bipolar spindle when Cin8 is clustered at the microtubule minus-end, and 2) for the maintenance of antiparallel microtubule cross-links in the spindle midzone. Our biochemical analysis of the Cin8 motor domain provides a detailed molecular model for how this clustering could occur to modulate Cin8's function *in vivo*.

## Experimental procedures

### Experimental conditions

Experiments were conducted at 298 K in ATPase buffer (20 mm HEPES, pH 7.2, using sodium hydroxide, 2 mm magnesium chloride, 20% (v/v) glycerol) unless otherwise noted. Typically, potassium chloride at 7.5 mm (referred to as low ionic strength) or 75 mm (referred to as high ionic strength) was added to the ATPase buffer. For ionic strength determination, the buffer contributed 7 mm and magnesium chloride contributed 6 mm plus the molarity of the monovalent potassium chloride added to the ATPase buffer. For biochemistry experiments using microtubules, 20 μm Taxol (paclitaxel) was added to the ATPase buffer to stabilize microtubule polymers.

### Cloning

DNA constructs coding for the motor domain of *S. cerevisiae* kinesin-5 Cin8 (residues 70–535) were synthesized by polymerase chain reaction (PCR) using *S. cerevisiae* genomic DNA as the template (generous gift from Soni Lacefield) and the oligonucleotides 5′-TCTAGAGGATCCAATGAGGAACTGAACATCACTGTAGC-3′ and 5′-TCTAGACTCGAGTTAAGTTATATTTTTAACCAAAATATCCTTCATTATAAATGAACCC-3′. The recombinant genes were ligated into modified pET16b and pET32b *Escherichia coli* expression vectors (67). A construct containing the N-terminal sequence (residues 1–69) did not express in *E. coli* (data not shown). Because the N-terminal sequence was not required for Cin8's *in vivo* function (14), we expected our Cin8 constructs would still retain ATPase activity and microtubule binding. Cin8-His contained the Cin8 motor domain fused to N-terminal His_6_ and TEV cleavage tags (expected mass = 53.6 kDa). Cin8-trx contained the Cin8 motor domain fused to N-terminal thioredoxin (TrxA), His_6_, TEV cleavage, and Arg_9_ tags (expected mass = 67.3 kDa). Cin8-trxΔR was like Cin8-trx yet did not contain the Arg_9_ tag (expected mass = 65.6 kDa). The most prominent difference in the Cin8 kinesin motor domain is the 99-residue insert in loop 8 (residues 254–353). To investigate the role of this loop 8 insert in the microtubule-binding behavior of Cin8 motors, we purified a construct in which this 99-residue segment was substituted with the 7-residue loop 8 in the related *S. cerevisiae* kinesin-5 Kip1 (sequence NNNNNSS) as was done previously (14, 47). Double fusion PCR was used to generate Cin8-His-Δ99 and Cin8-trx-Δ99 (4) using the oligonucleotides 5′-GGATTTTTGATAATAACAACAATAATTCATCCATATACATCCAGAATTTGCAAGAATTTCACATAAC-3′ and 5′-GGATGTATATGGATGAATTATTGTTGTTATTATCAAAAATCCTCAATTTTTTCATAAATTGGCCG-3′. The DNA construct coding for the motor domain of human kinesin-5 (Eg5; residues 1–368) was cloned into a modified pET16b vector for bacterial expression to achieve an N-terminal His_6_ tag and TEV cleavage sequence (N terminus, MGSSHHHHHHSSGENLYFQGSH^1^MASQ). Mutant Eg5 (S233C/R234K; called Eg5-SCRK) contained two switch-1 substitutions, which abolishes ATP hydrolysis activity (68).

### Protein expression and purification

Each construct was cotransformed into B834(DE3) cells (EMD Millipore, Darmstadt, Germany) with pRILP (Agilent Technologies, Santa Clara, CA). Kinesin expression was induced with 0.4 mm isopropyl 1-thio-β-d-galactopyranoside, and cells were grown overnight (12–18 h) at 291 K. Kinesin was purified from a clarified cell lysate using SP-Sepharose (Sigma, Darmstadt, Germany) and nickel-nitrilotriacetic acid (Qiagen, Germantown, MD) chromatography. Eluted protein was concentrated and loaded onto a Sephadex G-25 (GE Healthcare) desalting column for buffer exchange into final buffer (20 mm HEPES, pH 7.2, with sodium hydroxide, 2 mm magnesium chloride, 7.5 mm potassium chloride, 2 mm dithiothreitol, 0.1 mm ATP, 10% (v/v) glycerol). The final protein concentration was 10–75 μm (∼2–10 mol of ADP per mol of kinesin). Expression and purification of nucleotide-free human kinesin-5 were performed as described previously (21, 22, 68, 69). Microtubules were prepared using an aliquot of purified bovine brain tubulin that was thawed and cycled, and the microtubules were stabilized with 50 μm paclitaxel (Thermo Fisher Scientific, Waltham, MA). Each kinesin preparation was 90–95% pure via densitometry analysis of Coomassie-stained SDS gels, with Cin8-His and Cin8-His-Δ99 showing evidence of minor degradation products (supplemental Fig. S1*A*). We removed purification tags from the Cin8 constructs using TEV protease to test native motor domain behavior in comparison with the tagged variants (supplemental Fig. S1*B*).

To remove the C-terminal tails of α- and β-tubulin, microtubules were treated with 200 μg ml^−1^ subtilisin for 1 h at 303 K as described previously (70). Proteolysis was stopped with the addition of 10 mm PMSF (Roche Diagnostics). Subtilisin-treated microtubules were spun at 100,000 × *g* for 30 min at 298 K and were resuspended in ATPase buffer, including 50 μm paclitaxel. Protein concentration was determined using Coomassie plus Bradford assay reagent (Thermo Fisher Scientific) with bovine serum albumin (BSA) as the standard as well as measuring absorbance at 280 nm for kinesin. The error in protein concentration was 14% for kinesin and 17% for tubulin.

### Analytical size-exclusion chromatography

Purified proteins were resolved by using a Superose 6 Increase 10/300 gel filtration column (GE Healthcare) at 293 K in 20 mm HEPES, pH 7.2, and 150 mm potassium chloride. The elution volumes (*V_e_*) of proteins were determined by optical absorbance (λ = 280 nm) with a constant flow rate of 0.5 ml/min. Excluded volume was estimated using blue dextrin (∼2 MDa; 2 mg/ml), and the included volume was determined by running 1 mm ATP.

### Steady-state ATPase assay

The basal and microtubule-stimulated ATPase activities of Cin8 were measured by the NADH coupled assay (71–75). Briefly, Cin8 or microtubule·Cin8 was diluted to 2× final concentration in ATPase buffer. ATP was also diluted to 2× final concentration in ATPase buffer plus 2× KCl and 2× NADH mixture (final concentrations: 0.5 mm phosphoenolpyruvate, 0.4 mm NADH, 5 units/ml rabbit pyruvate kinase (Roche Diagnostics), 8 units/ml lactic dehydrogenase (Sigma, Darmstadt, Germany)). To initiate the reaction, equal volumes (20 μl) of Cin8 or microtubule·Cin8 and ATP, KCl, and NADH mixture were thoroughly mixed by pipetting, and 30 μl of each reaction was transferred to a 384-well microplate for absorbance reading at 340 nm using a microplate spectrophotometer (BioTek, Winooski, VT). A standard curve from 0 to 800 μm NADH was used to convert *A*_340_ to ADP product concentration. For ATP-dependent experiments, the initial ATPase velocity was plotted against the ATP concentration, and the data were fit to the Michaelis-Menten Equation 1,

(Eq. 1) $$v_{\text{o}}=\frac{\left[E_{\text{o}}\right]k_{\text{cat}}\left[\text{ATP}\right]}{K_{m,\text{ATP}}+\left[\text{ATP}\right]}$$

where [*E*_o_] is the total concentration of Cin8; *k*_cat_ is the maximum rate of ATP turnover per Cin8 active site at infinite [ATP], and *K_m_*_, ATP_ is the Michaelis constant, which is defined as the [ATP] that yields ½[*E*_o_]*k*_cat_.

For microtubule-dependent experiments, the initial ATPase velocity was plotted against the microtubule concentration, and the data were fit to the quadratic Equation 2,

(Eq. 2) vo=kcat{([Eo]+K0.5,MT+[MT])−([Eo]+K0.5,MT+[MT])2−4[Eo][MT]2}+kbasal

where [*E*_o_] is the total Cin8 concentration; *k*_cat_ is the maximum rate of ATP turnover per Cin8 active site at infinite microtubule concentration; *K*_0.5, MT_ is the microtubule concentration that yields ½[*E*_o_]*k*_cat_, and *k*_basal_ is the experimentally determined basal rate of ATP turnover at zero microtubule concentration. Each plot corresponds to the average ATPase rate normalized to 1 μm motor for *n* = 5–8 experiments with error bars showing the standard deviation of each data point when replicate experiments were performed at high ionic strength.

### Cosedimentation assays

Experiments were performed, as described previously (76), where the purified Cin8 with no added nucleotide was incubated with paclitaxel-stabilized microtubules for 15–60 min, and microtubule·Cin8 complexes were pelleted by centrifugation in a Beckman Optima L-100 XP ultracentrifuge using the Ti-42.2 fixed-angle rotor at 100,000 × *g* for 30 min at 298 K. For Cin8-trx, Cin8-trxΔR, and Cin8-Δ99, gel samples were prepared for the supernatant and pellet fractions at equal volumes, and the proteins were resolved by SDS-PAGE and stained with Coomassie Brilliant Blue R-250. The fraction of total Cin8 bound to microtubules was plotted against the microtubule concentration, and the data were fit to quadratic Equation 3,

(Eq. 3) $$f_{b}=\frac{\left(\left[E_{\text{o}}\right]+K_{d,\text{app}}+\left(s\left[\text{MT}\right]\right)\right)-\sqrt{{\left(\left[E_{\text{o}}\right]+K_{d,\text{app}}+\left(s\left[\text{MT}\right]\right)\right)}^{2}-4\left[E_{\text{o}}\right]\left(s\left[\text{MT}\right]\right)}}{2\left[E_{\text{o}}\right]}$$

where [*E*_o_] is the total Cin8 concentration; *K_d_*_, app_ is the apparent *K_d_*_, MT_ that represents the average binding constant for all binding sites, and *s* is the stoichiometry coefficient to quantify the number of binding sites. Experiments with Eg5 and Eg5-SCRK were performed side-by-side using nearly identical microtubule concentrations to control for error in microtubule concentration determination. Each plot corresponds to the average fraction bound at each individual molar ratio (microtubule/Cin8 or microtubule/Eg5) for *n* = 3–7 experiments, with error bars showing the standard deviation of each data point.

For cosedimentation experiments with Cin8-His and Cin8-tagless, which cannot be resolved from tubulin on an SDS gel due to its comparable molecular weight, the quantity of Cin8-His in the supernatant was measured using the NADH coupled assay. A supernatant sample was mixed with an equal volume of 2× NADH reaction mix (1× concentrations: 0.5 mm phosphoenolpyruvate, 0.4 mm NADH, 5 units/ml rabbit pyruvate kinase, 8 units/ml lactic dehydrogenase, 1 mm ATP, 4 μm tubulin as microtubules). The fraction of Cin8-His bound (*f_b_*) at each condition was determined by Equation 4.

(Eq. 4) $$f_{b}=1-\left(\frac{\text{supn}\;\text{ATPase}\;\text{rate}\;\text{with}\;\text{increasing}\;\left[\text{MT}\right]}{\text{supn}\;\text{ATPase}\;\text{rate}\;\text{with}\;0\;\left[\text{MT}\right]}\right)$$

### Competitive cosedimentation experiments

A series of competition experiments were designed to characterize the microtubule-binding behavior of Cin8 compared with Eg5. For experiments where both Eg5 and Cin8 were incubated with microtubules in the same reaction, the mixture was equilibrated at 298 K for 60 min before centrifugation. Data were fit to Equation 5 (77),

(Eq. 5) $$f_{b}^{\text{Eg}5}=\frac{\left[\text{Eg}5\right]}{K_{d,\text{Eg}5}\left(1+\frac{\left[\text{Cin}8\right]}{K_{d,\text{Cin}8}}\right)+\left[\text{Eg}5\right]}$$

where *f*_*b*_^Eg5^ is the fraction of Eg5 bound to the microtubules; [Eg5] and [Cin8] are the total Eg5 and Cin8 concentrations (respectively); and *K_d_*_, Eg5_ and *K_d_*_, Cin8_ are the apparent microtubule-binding affinities for Eg5 and Cin8, respectively. The magnitude of *K_d_*_, Eg5_ was set to 0.0007 μm at low ionic strength and 0.501 μm at high ionic strength during the nonlinear least squares fitting. Error bars correspond to the standard deviation obtained from *n* = 3–6 experiments.

### Presteady-state kinetic experiments

Stopped-flow measurements were performed at 298 K using an SF-300X stopped-flow instrument (KinTek, Snow Shoe, PA) equipped with a xenon arc lamp (Hamamatsu, Hamamatsu City, Japan). The kinetics of Cin8 and Eg5 association with microtubules were determined by monitoring the change in solution light scattering (λ = 340 nm at 90° to the incident beam). Transients were fit to a single exponential function, and the amplitudes and observed rates (*k*_obs_) were plotted as a function of Cin8 concentration. Amplitude data were fit to hyperbolic Equation 6,

(Eq. 6) $$\text{amplitude}=\frac{A_{\max}\left[\text{Cin}8\right]}{K_{0.5}+\left[\text{Cin}8\right]}$$

where *A*_max_ is the maximum extrapolated amplitude at infinite [Cin8], and *K*_0.5_ is the [Cin8] that yields ½*A*_max_. Observed rate plots showed a minimum model for two-step binding kinetics based on Equation 7,

(Eq. 7) $$\text{Cin}8+\text{MT}\overset{K_{+1}}{⇌}\;\text{Cin}8\cdot \text{MT}\overset{k_{+2}}{\underset{{\text{k}}_{-2}}{⇌}}\;\text{Cin}8*\cdot \text{MT}$$

and data were fit to linear Equation 8,

(Eq. 8) $$k_{\text{obs}}=K_{+1}k_{+2}\left[\text{Cin}8\right]+k_{-2}$$

where *k*_obs_ is the observed exponential rate constant for the change in light scattering; *K*_+1_*k*_+2_ is the apparent second-order rate constant for Cin8 association with microtubules, and *k*_−2_ corresponds to the observed rate constant for motor dissociation from the microtubule·Cin8 complex as determined from the *y*-intercept.

### Reversibility test for Cin8 aggregation on the microtubule

We designed an experiment to test whether Cin8 oligomerization on the microtubule under super-stoichiometric conditions was reversible. Two reactions were set up for side-by-side comparison. The first tube contained 6 μm motor plus 0.5 μm microtubules (12:1 super-stoichiometry), and the second control tube contained only a 6 μm motor, and both were pre-equilibrated for 30 min at room temperature. After the 30-min pre-equilibration, each tube was diluted 10-fold using the same reaction mixture bringing the final concentrations for the ATPase assay at 0.6 μm motor, 6.1 μm microtubules, 1 mm ATP, 8.5 mm KCl, NADH mixture. These reactions were transferred to a microplate for absorbance reading at 340 nm.

### Cryo-EM sample preparation

Bovine tubulin (Cytoskeleton, Denver, CO) was resuspended in SB buffer (25 mm PIPES, pH 6.8, with sodium hydroxide, 25 mm sodium chloride, 1 mm EGTA, 2 mm magnesium chloride) to 10 mg/ml with 2 mm GTP added. After clarification in a prechilled Beckman Optima TL100 ultracentrifuge and TLA120.2 fixed angle rotor (100,000 rpm, 10 min, 277 K), the supernatant was immediately incubated at 310 K. After 10 min of polymerization, a diluted Taxol solution (200 μm paclitaxel in SB buffer) was added and incubated at 310 K for an additional 30–45 min to allow microtubules to polymerize. Microtubules were pelleted through a glycerol cushion (50 μl of 60% (v/v) glycerol in SB buffer) using the same ultracentrifuge equipment (50,000 rpm, 20 min, 297 K). The pellet was washed twice and resuspended to 15-μl final volume (∼20 μm microtubules) using SB buffer containing ∼20 μm paclitaxel.

To remove glycerol and reduce the ionic strength to promote microtubule complex formation, Cin8-His was buffer-exchanged for several rounds using an Amicon Ultra (10,000 MWCO, 0.5 ml) centrifugal filter into SB buffer containing 2 mm dithiothreitol, 2 mm ATP, 2 mm aluminum chloride, and 10 mm sodium fluoride. To form the complex, Cin8 and microtubules were separately diluted in water and mixed to give a final microtubule concentration of 2 μm and ∼0.9× molar ratio of Cin8 to prevent microtubule bundling at higher ratios. After 5 min of incubation at room temperature, 4 μl of this mixture was added to a holey carbon grid with 1.2-μm hole diameter (Quantifoil) without glow discharging to maximize Cin8 decoration. After an initial wicking step to remove most of the solution, the grid was completely blotted manually and immediately plunged into liquid ethane in a home-built manual plunger. To achieve the no-nucleotide state, *Solanum tuberosum* apyrase (Grade VII; Sigma, Darmstadt, Germany) was added after complex formation and allowed to incubate for 30 min until all ATP or ADP was cleaved to AMP.

### Cryo-EM data collection and image processing

Micrographs were collected using SerialEM on a Tecnai F20 electron microscope (FEI, Waltham, MA) operated at 200 kV with a K2 Summit direct electron detector (Gatan, Pleasanton, CA) in counting mode with dose fractionation. The nominal magnification was ×27,000, resulting in a final pixel size of 1.247 Å on the specimen level. Thirty five frames of 300 ms each were collected at a dose rate of 9.3 e^−^ pixel^−1^ s^−1^ with a total dose of 60 e^−^/Å^2^. Defocus values ranging from −0.8 to −3.0 μm were used. After the movie frames were aligned using the Unblur program (78), overlapping boxed segments were manually picked from individual microtubules. Defocus and astigmatism parameters were estimated using CTFFIND3 (79).

Three-dimensional (3D) helical reconstruction was performed as described previously (58). Initial reference models for 12-, 13-, and 14-protofilament microtubules were generated by applying a parametric model (80) to PDB models of the no-nucleotide kinesin·microtubule complex (58, 81) and applying a low-pass filter with a 20-Å cutoff. Because 13-protofilament microtubules were the most common (∼60% total selected filaments) this sub-population was chosen for further analysis. Initial estimation of in-plane rotation of individual segments and semi-exhaustive searching of *XY* shifts/Euler angles was performed using the Radon transform (82) and SPIDER package (83), respectively. The filament polarity and location of the microtubule seam was determined using reference alignment, followed by local refinement of every segment using the established seam orientation and polarity. A final round of SPIDER refinement was performed following refinement of the Euler angles and shifts for each box. The final data set contained exactly one box for each 8-nm repeat identified in the selected microtubules. Three iterative rounds of FREALIGN refinement and three-dimensional reconstruction were performed using successively higher resolution cutoffs (20, 15, and 12 Å) with a FREALIGN score threshold of 27. Because of the misalignment between α- and β-tubulin at the microtubule seam, application of pseudo-helical symmetry results in a microtubule reconstruction that contains only one “good” protofilament (58, 59, 81). After applying 13-fold pseudo-helical symmetry, this single good protofilament was extracted from the reconstruction using a wedge-shaped three-dimensional mask and then was replicated and transformed 13 times using refined helical parameters to generate the final asymmetric microtubule model. For the final ∼7-Å reconstruction of Cin8, a total of 2282 particles (8-nm repeats) was used, for a total of 27,547 asymmetric units (13-protofilament). The cryo-EM map for the ADP·AlF*_x_* state of the microtubule·Cin8 complex has been deposited in the EMDB (accession code EMD-8408).

### Primary structure analysis for Cin8 intrinsically disordered regions

Eight different algorithms were used to predict intrinsically disordered regions of the Cin8 motor domain plus the N-terminal extension (Cin8 residues 1–535): PONDR (VL3, VL-XT, and XL1-XT (84–91)); disEMBL (92); RONN (93); IUPred (94, 95); DisProt (96); and PrDOS (97). Each algorithm provided a numerical score (0–1) for each residue position with scores greater than 0.5 considered as disordered. Scores were averaged at each residue, and the standard deviation of the mean residue score was calculated.

### MSE analysis

Cosedimentation binding curves were fit to quadratic Equation 2 allowing both *K_d_*_, app_ and *s* to float during nonlinear least squares fitting in Mathematica (Wolfram, version 10). The overall minimum MSE (MSE_min_) was determined by fixing *s* and floating *K_d_*_, app_. MSE_min_ was divided by the MSE observed at each fixed *s* value (MSE_fit_), and the resulting curve was interpolated to provide the asymmetric confidence interval for *s* based on a 25% increase in MSE (*i.e.* MSE_min_/MSE_fit_ (98)).

## Author contributions

K. M. B., H. K. C., C. V. S., and J. C. C. designed the study. K. M. B., H. K. C., C. V. S., and J. C. C. performed the research. K. M. B., H. K. C., C. V. S., and J. C. C. wrote the manuscript.

## Supplementary Material

Supplemental Data

## Footnotes

2 The abbreviations used are:

AMPPNP: adenosine 5′-β,γ-imidotriphosphate
MSE: mean square error
TEV: tobacco etch virus
PDB: Protein Data Bank.
