# Supplementary material for: The yeast kinesin-5 Cin8 interacts with the microtubule in a noncanonical manner
Source: J Biol Chem. 2017 Jul 12;292(35):14680–94. doi: 10.1074/jbc.M117.797662 (PMC5582858; doi:10.1074/jbc.M117.797662)
Supplement: Supplemental Data [file supp_292_35_14680__index.html]

The yeast kinesin-5 Cin8 interacts with the microtubule in a noncanonical manner — The yeast kinesin-5 Cin8 interacts with the microtubule in a noncanonical manner — Noncanonical microtubule interaction by Cin8 — Supplemental Data 

# The yeast kinesin-5 Cin8 interacts with the microtubule in a noncanonical manner

## Supplemental Data

- Supplemental Data (.pdf, 6.7 MB) - Supplemental data file
