# Supplementary material for: The yeast kinesin-5 Cin8 interacts with the microtubule in a noncanonical manner
Source: J Biol Chem. 2017 Jul 12;292(35):14680–94. doi: 10.1074/jbc.M117.797662 (PMC5582858; doi:10.1074/jbc.M117.797662)
Supplement: Supplemental Data [file 10.1074_M117.797662_jbc.M117.797662-1.pdf]

SUPPLEMENTAL INFORMATION

The yeast kinesin-5 Cin8 interacts with the microtubule in a noncanonical manner

Kayla M. Bell[1], Hyo Keun Cha[2], Charles V. Sindelar[3], Jared C. Cochran[1,*]

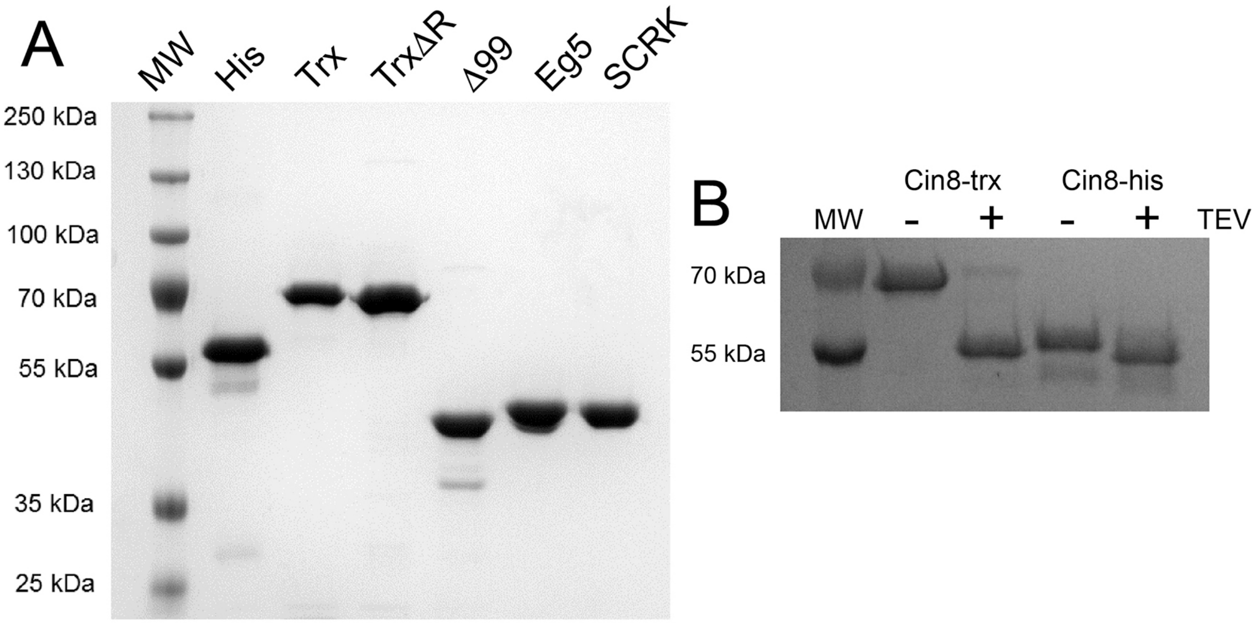

Figure S1. **Purified kinesin-5 motors.** (***A***) Cin8 and Eg5 proteins were electrophoresed using a 10% SDS polyacrylamide gel as indicated. (***B***) To remove all purification tags N-terminal to the TEV cleavage site, both Cin8-his and Cin8-trx were incubated with TEV protease (5:1 Cin8:TEV) for 60 min at room temperature. A 10% SDS polyacrylamide gel showing TEV cleavage of purification tags.

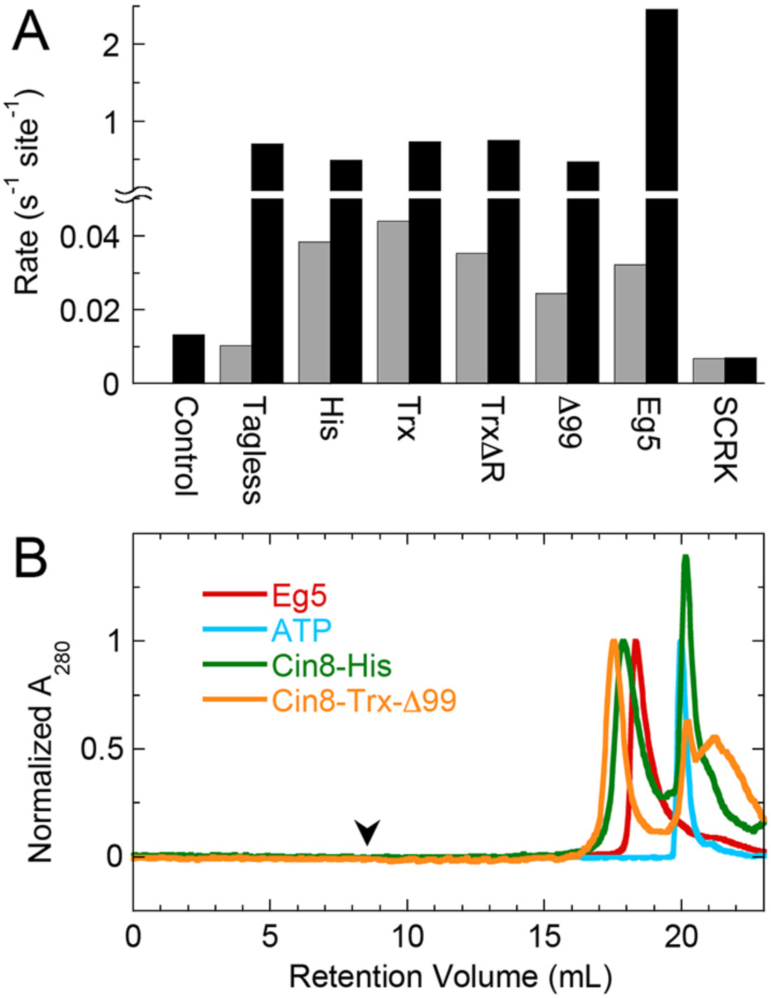

Figure S2. **Kinesin-5 motors are monomeric with microtubule-stimulated ATPase activity.** (***A***) Basal (gray) and microtubule-stimulated (black) ATPase activity of kinesin-5 motors at low ionic strength using the NADH coupled assay. Rates were normalized to 1 μM motor. Final concentrations: 1 mM ATP, 4 μM microtubules. (***B***) Superose-6 gel filtration chromatograms show normalized absorbance (λ = 280 nm) versus retention volume for Eg5 (red), ATP only (blue), Cin8-his (green), and Cin8-trx-Δ99 (orange). Arrow indicates blue dextrin (~2 MDa) retention.

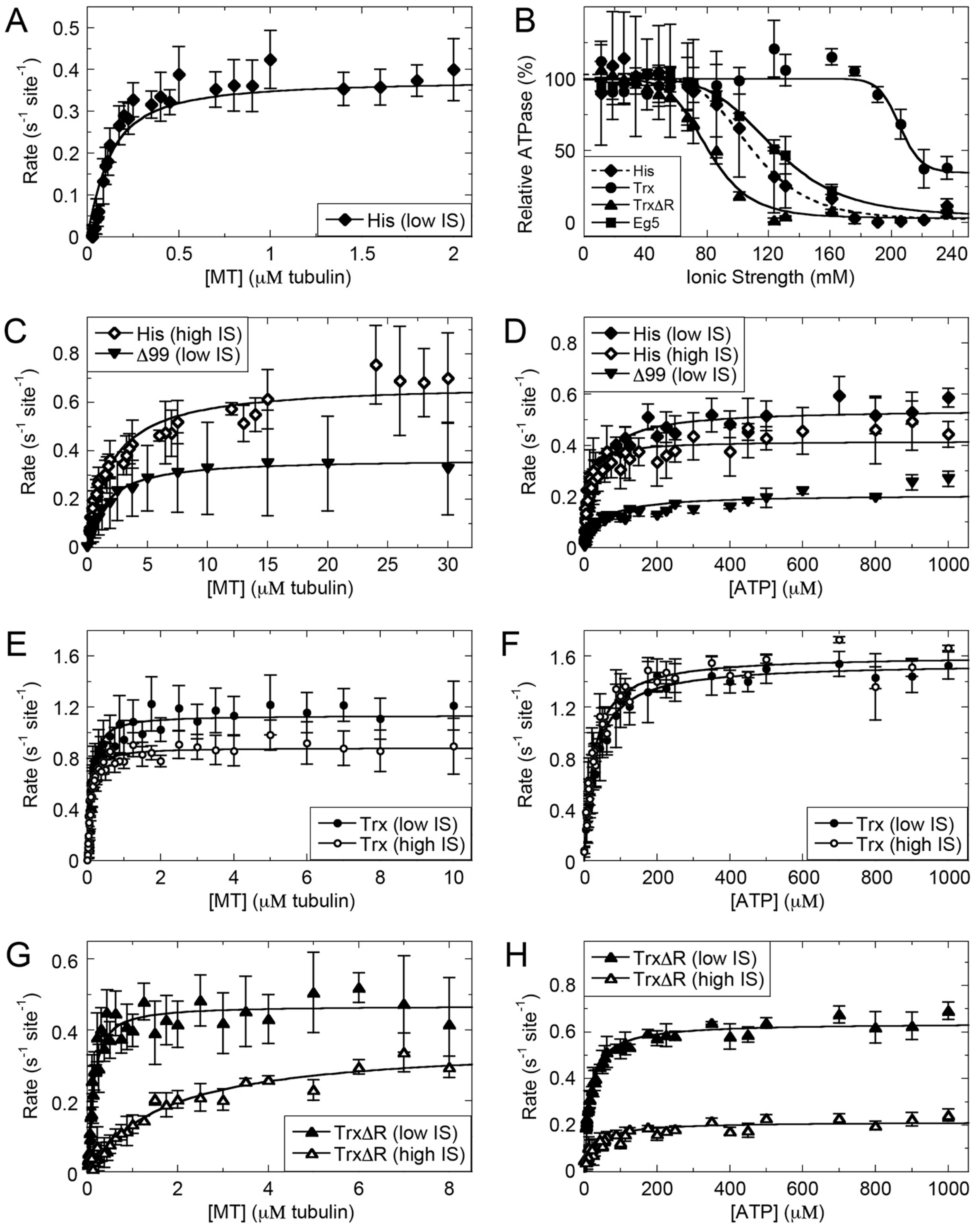

Figure S3. (legend on the next page)

Figure S3. **Steady state kinetics of Cin8 motors reveals ionic strength-dependence of microtubule binding affinity and ionic strength-independence of ATP binding affinity.** Each plot corresponds to the average ATPase rate normalized to 1 μM motor for n = 3-5 experiments with error bars showing the standard deviation of each data point. (***A***) Microtubule-stimulated ATPase activity for Cin8-his at low ionic strength. Final concentrations: 0.01 μM Cin8, 0–2 μM microtubules, 1 mM ATP. Data were fit to the equation 2 to yield k_{cat} at 0.38 ± 0.01 s^{-1} $site^{-1}$ and $K_{0.5,\mathrm{MT}}$ = 0.08 ± 0.02 μM. (***B***) Relative ATPase activity for Cin8-trx (circles), Cin8-his (diamonds), Cin8-trxΔR (triangles), and Eg5 (squares) as a function of ionic strength. Final concentrations: 0.5 μM Cin8 or 0.2 μM Eg5, 2 μM microtubules, 1 mM ATP, 7.5–225 mM KCl. (***C***) Microtubule-stimulated ATPase activity for Cin8-his (open diamonds) at high ionic strength and Cin8-his-Δ99 (closed triangles) at low ionic strength. Final concentrations: 0.2 μM Cin8-his, 0.5 μM Cin8-his-Δ99, 0–30 μM microtubules, 1 mM ATP. Cin8-his: k_{cat} = 0.68 ± 0.02 s^{-1} $site^{-1}$, $K_{0.5,\mathrm{MT}}$ = 1.8 ± 0.2 μM. Cin8-his-Δ99: k_{cat} = 0.37 ± 0.01 s^{-1} $site^{-1}$, $K_{0.5,\mathrm{MT}}$ = 1.3 ± 0.1 μM. (***D***) ATP-stimulated ATPase activity for Cin8-his at low ionic strength(closed diamonds), Cin8-his at high ionic strength(open diamonds), and Cin8-his-Δ99 at low ionic strength(closed triangles). Final concentrations: 0.1 μM Cin8-his, 0.5 μM Cin8-his-Δ99, 2 μM microtubules, 5–1000 μM ATP. Data were fit to equation 1. Cin8-his at low ionic strength: k_{cat} = 0.54 ± 0.01 s^{-1} $site^{-1}$, $K_{m,\mathrm{ATP}}$ = 29 ± 2 μM. Cin8-his at high ionic strength: k_{cat} = 0.42 ± 0.01 s^{-1} $site^{-1}$, $K_{m,\mathrm{ATP}}$ = 12 ± 1 μM. Cin8-his-Δ99: k_{cat} = 0.21 ± 0.01 s^{-1} $site^{-1}$, $K_{m,\mathrm{ATP}}$ = 45 ± 8 μM. (***E***) Microtubule-stimulated ATPase activity of Cin8-trx at low (closed circles) and high ionic strength(open circles). Final concentrations: 0.1 μM Cin8, 0–10 μM microtubules, 1 mM ATP. Cin8-trx at low ionic strength: k_{cat} = 1.1 ± 0.02 s^{-1} $site^{-1}$, $K_{0.5,\mathrm{MT}}$ = 0.07 ± 0.01 μM. Cin8-trx at high ionic strength: k_{cat} = 0.88 ± 0.01 s^{-1} $site^{-1}$, $K_{0.5,\mathrm{MT}}$ = 0.05 ± 0.01 μM. (***F***) ATP-stimulated ATPase activity of Cin8-trx at low (closed circles) and high ionic strength(open circles). Final concentrations: 0.2 μM Cin8, 2 μM microtubules, 5–1000 μM ATP. Cin8-trx at low ionic strength: k_{cat} = 1.5 ± 0.02 s^{-1} $site^{-1}$, $K_{m,\mathrm{ATP}}$ = 29 ± 2 μM. Cin8-trx at high ionic strength: k_{cat} = 1.6 ± 0.03 s^{-1} $site^{-1}$, $K_{m,\mathrm{ATP}}$ = 25 ± 2 μM. (***G***) Microtubule-stimulated ATPase activity of Cin8-trxΔR at low (closed triangles) and high ionic strength(open triangles). Final concentrations: 0.1 μM Cin8, 0–10 μM microtubules, 1 mM ATP. Cin8-trxΔR at low ionic strength: k_{cat} = 0.47 ± 0.01 s^{-1} $site^{-1}$, $K_{0.5,\mathrm{MT}}$ = 0.08 ± 0.01 μM. Cin8-trxΔR at high ionic strength: k_{cat} = 0.36 ± 0.02 s^{-1} $site^{-1}$, $K_{0.5,\mathrm{MT}}$ = 1.7 ± 0.2 μM. (***H***) ATP-stimulated ATPase activity of Cin8-trxΔR at low (closed triangles) and high ionic strength(open triangles). Final concentrations: 0.2 μM Cin8, 2 μM microtubules, 5–1000 μM ATP. Cin8-trxΔR at low ionic strength: k_{cat} = 0.64 ± 0.01 s^{-1} $site^{-1}$, $K_{m,\mathrm{ATP}}$ = 16 ± 1 μM. Cin8-trxΔR at high ionic strength: k_{cat} = 0.21 ± 0.01 s^{-1} $site^{-1}$, $K_{m,\mathrm{ATP}}$ = 29 ± 4 μM.

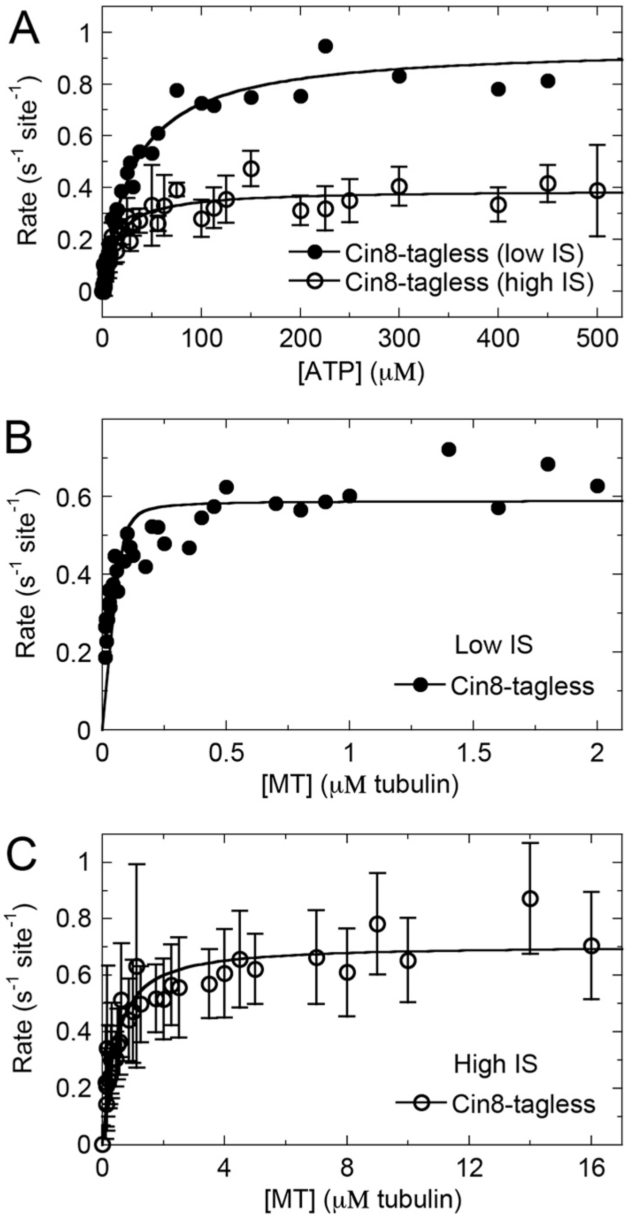

Figure S4. **Steady state kinetics of Cin8-tagless motors.** (***A***) ATP-stimulated ATPase activity for Cin8-tagless at low (closed circles) and high (open circles) ionic strength. Final concentrations: 0.1 μM Cin8, 2 μM microtubules, 5–1000 μM ATP. Data were fit to equation 1. Low: k_{cat} = 0.95 ± 0.02 s^{-1} $site^{-1}$ and $K_{m,ATP}$ = 31 ± 3 μM. High: k_{cat} = 0.39± 0.01 s^{-1} $site^{-1}$ and $K_{m,ATP}$ = 13 ± 2 μM. (***B***) Microtubule-stimulated ATPase activity for Cin8-tagless at low ionic strength. Final concentrations: 0.01 μM Cin8, 0–2 μM microtubules, 1 mM ATP. Data were fit to equation 2 to yield k_{cat} at 0.59 ± 0.03 s^{-1} $site^{-1}$ and $K_{0.5,MT}$ = 0.004 ± 0.004 μM. (***C***) Microtubule-stimulated ATPase activity for Cin8-tagless at high ionic strength. Final concentrations: 0.2 μM Cin8, 0–30 μM microtubules, 1 mM ATP. Data were fit to equation 2 to yield k_{cat} at 0.71 ± 0.02 s^{-1} $site^{-1}$ and $K_{0.5,MT}$ = 0.33 ± 0.06 μM.

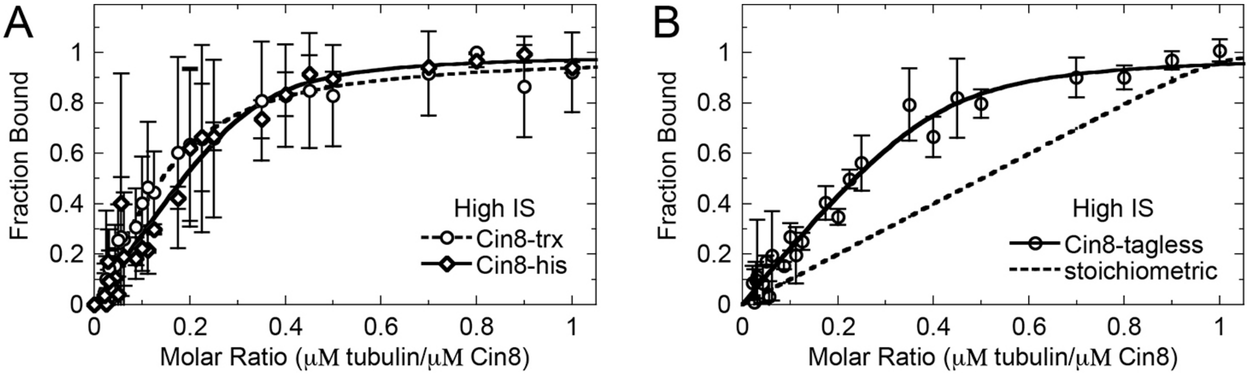

Figure S5. **Cosedimentation assays demonstrate superstoichiometric Cin8-his and Cin8-tagless interaction with microtubules at high ionic strength.** (***A***) Cin8-trx (circles, dashed line) and Cin8-his (diamonds, solid line) cosedimentation results using the NADH coupled assay to quantify soluble Cin8-his as a function of microtubule concentration. Final concentrations: 2 μM Cin8, 0–2 μM microtubules. Cin8-trx: $K_{d,app}$ = 0.32 ± 0.11 μM and s = 5.8 ± 0.9. Cin8-his: $K_{d,app}$ = 0.06 ± 0.04 μM and s = 3.0 ± 0.3. (***B***) Cin8-tagless cosedimentation results using the NADH coupled assay at high ionic strength. Final concentrations: 2 μM Cin8, 0–2 μM microtubules. $K_{d,app}$ = 0.08 ± 0.03 μM and s = 2.4 ± 0.2.

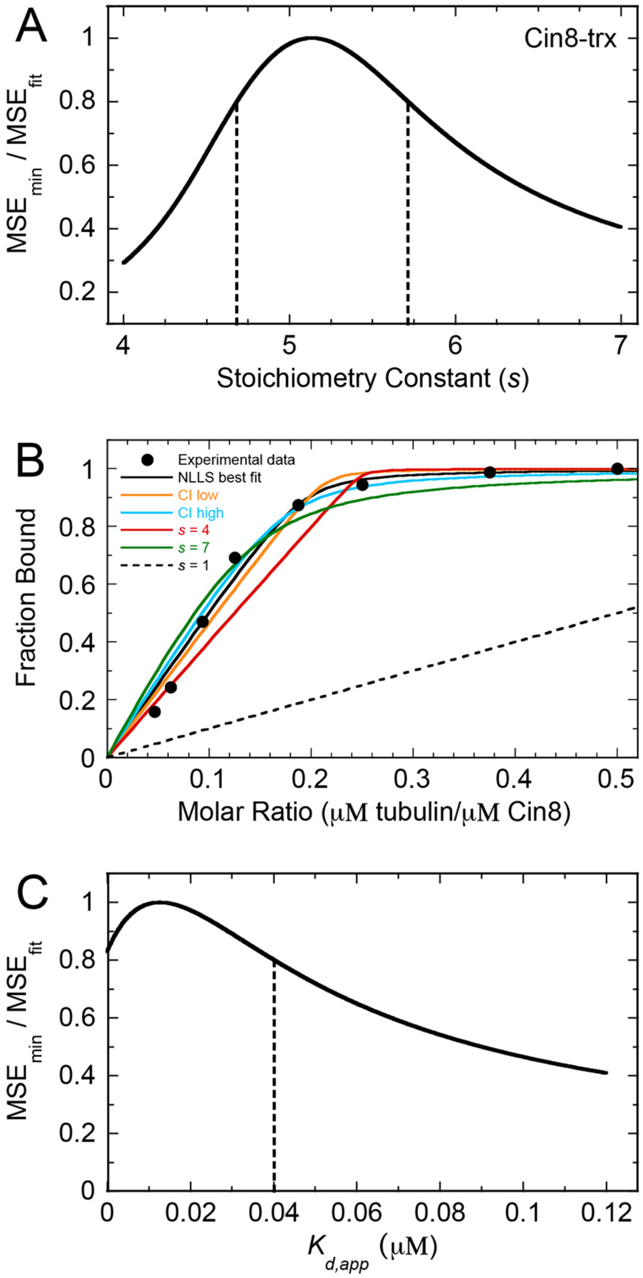

Figure S6. **MSE analysis defines asymmetric confidence intervals for equilibrium binding constant ($K_{d,app}$) and stoichiometry constant (*s*).** (***A***) Confidence plot for the fit of cosedimentation data to equation 3 for Cin8-trx binding microtubules (Fig. 2a) while the stoichiometry constant was fixed at different values (*s* = 4–7) and $K_{d,app}$ was floated in the nonlinear least squares (NLLS) fitting analysis. MSE_{min} corresponds to the MSE calculated when the stoichiometry constant was fixed at the best fit *s* value at 5.14 and $K_{d,app}$ was floated. Dashed lines correspond to the *s* values where the MSE_{min}/MSE_{fit} ratio drops to 0.8 (i.e. 25% increase in MSE_{fit}) to define the asymmetric confidence interval (CI_{low} = 4.68; CI_{high} = 5.71). (***B***) Cosedimentation data for Cin8-trx from Fig. 2a showing different curve fits (as indicated). (***C***) Confidence plot for the fit of cosedimentation data (Fig. 2a) while $K_{d,app}$ was fixed at different values ($K_{d,app}$ = 1x10^{-12}– 0.12) and *s* was floated. Best fit $K_{d,app}$ was 0.013 μM. Lower limit of the $K_{d,app}$ confidence interval was indistinguishable from zero while upper limit of $K_{d,app}$ was 0.04 μM (dashed line).

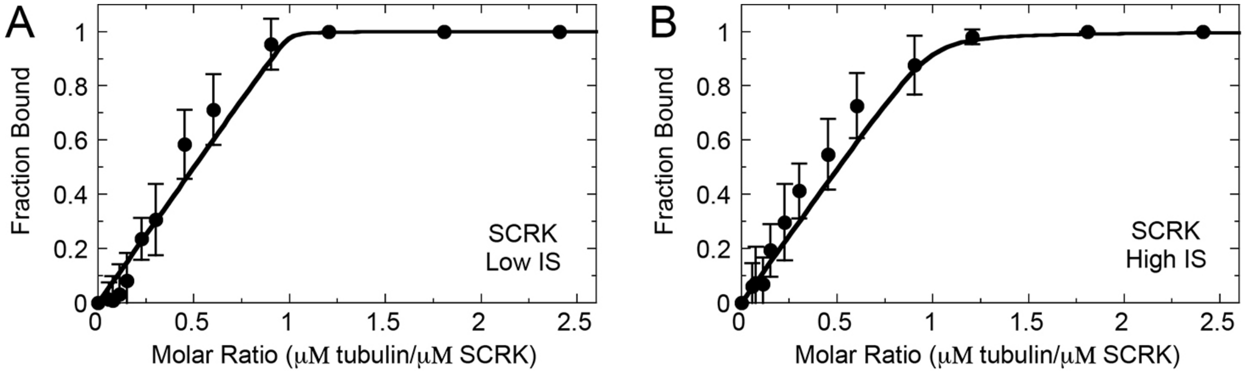

Figure S7. **Cosedimentation assays demonstrate tight, stoichiometric Eg5 SCRK interaction with microtubules.** Cosedimentation assays were used to monitor Eg5 SCRK binding to microtubules at low (***A***) and high (***B***) ionic strength (IS). Each plot corresponds to the average fraction bound at each individual molar ratio for n = 6 experiments with error bars showing the standard deviation of each data point. Final concentrations: 2 μM SCRK, 0–4.8 μM microtubules. The data were fit to equation 3 allowing the $K_{d,MT}$ constant to float in the analysis with $s = 1$. Low: $K_{d,app} = 0.0006 \pm 0.0009$ μM. High: $K_{d,app} = 0.008 \pm 0.010$ μM.

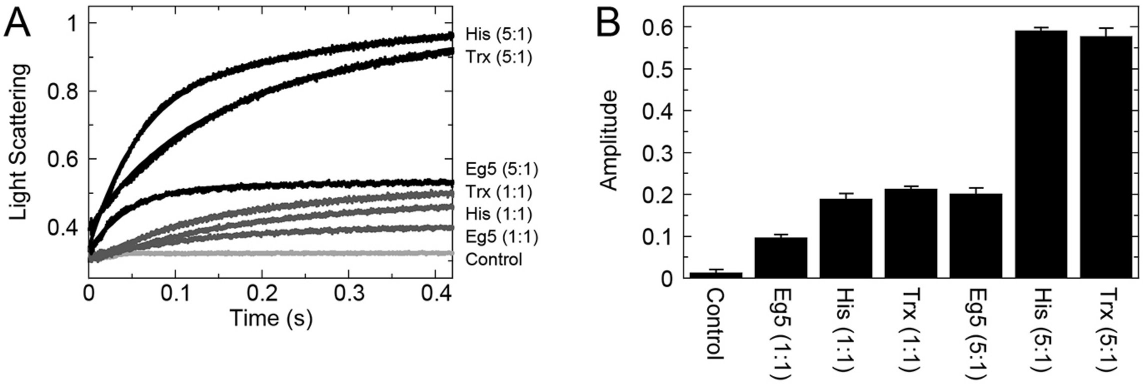

Figure S8. **Full transients for presteady-state kinetics of kinesin-5 binding microtubules.** (***A***) Stopped-flow transients showing the change in light scattering as Cin8 or Eg5 were rapidly mixed with microtubules under stoichiometric (1:1) and super-stoichiometric (5:1) conditions at low ionic strength. Final concentrations: 0.5 μM or 2.5 μM Cin8-trx, Cin8-his or Eg5, 0.5 μM microtubules. Control reaction was light scattering from microtubules alone. (***B***) Bar graph showing the average transient amplitudes from panel *A* (n = 5–7 transients). Error bars denote the standard deviation of the mean.

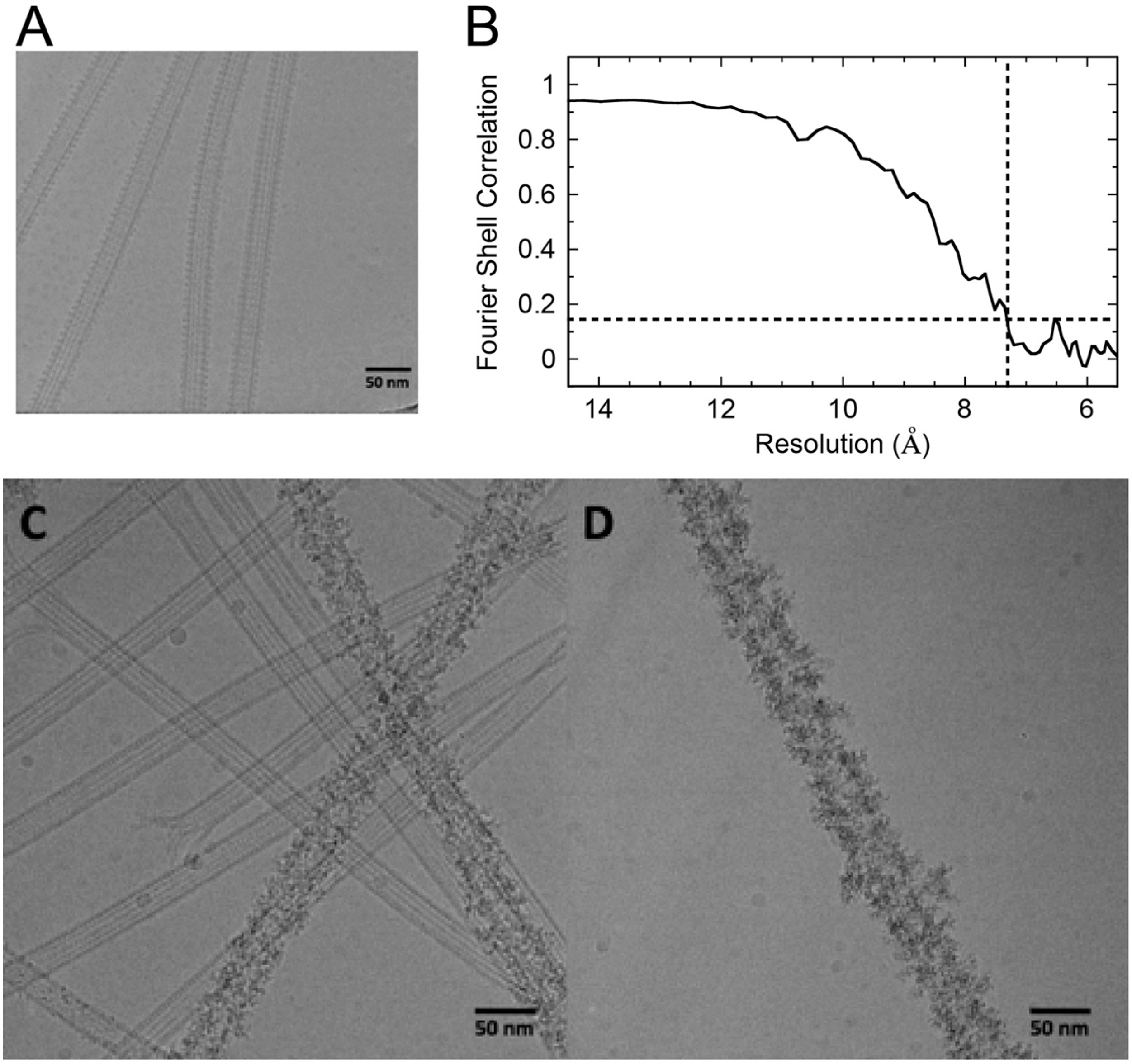

Figure S9. **Cryo-EM micrographs of stoichiometric and superstoichiometric Cin8•microtubule complexes and Fourier shell correlation (FSC) curve for the reconstruction in ADP•AlF$_x$ state.** (***A***) Images of Cin8-decorated microtubules in 1:1 stoichiometric state at 27,000x magnification. (***B***) A final resolution of ~7.3Å was estimated by calculating a FSC between two independent half maps and using the FSC criterion of 0.143, which is indicated by the horizontal dotted line. (***C***) Cooperative, super-stoichiometric decoration of microtubules by Cin8 in the no-nucleotide state after apyrase treatment. (***D***) Example of a relatively thicker filament with super-stoichiometric levels of Cin8 binding with 0.5x molar ratio added.

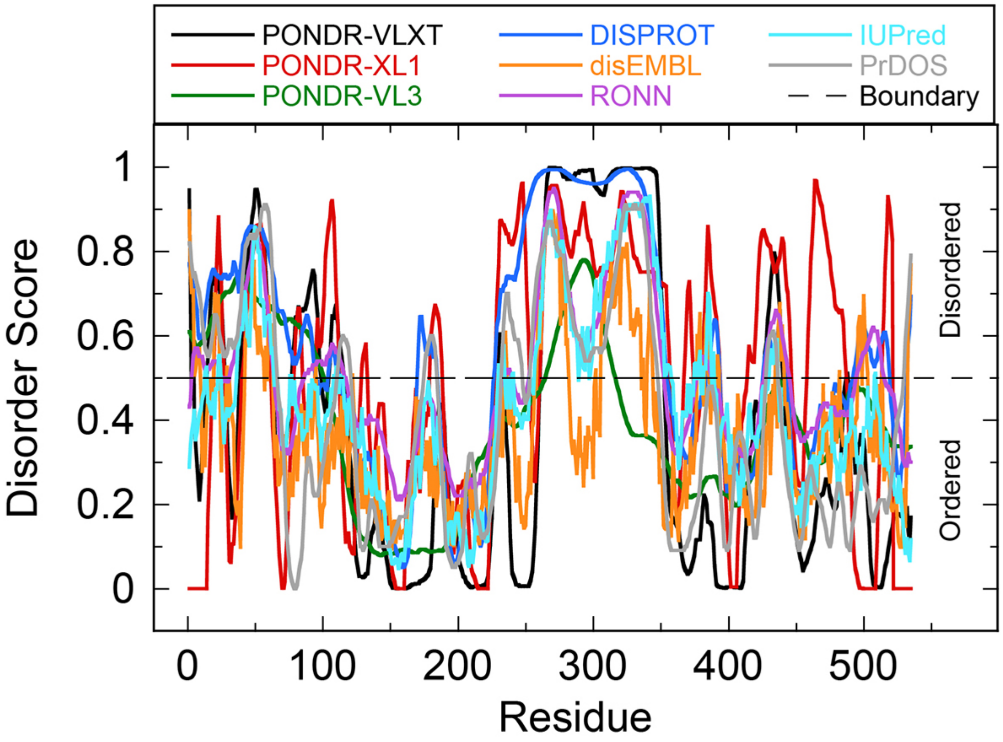

Figure S10. **Cin8 primary structure analysis demonstrates intrinsically disordered loop 8.** Raw prediction scores for Cin8 (residues 1–535) based on eight different algorithms. Boundary indicates the threshold for disorder prediction.

Table S1. **Steady-state constants for various kinesin-5 proteins.**

	**Eg5** [a]	**Cin8-tagless**		**Cin8-his**		**Cin8-trx**		**Cin8-trxΔR**		**Δ99**
**Ionic strength:**	**21 mM**	**21 mM**	**88 mM**	**21 mM**	**88 mM**	**21 mM**	**88 mM**	**21 mM**	**88 mM**	**21 mM**
k_{cat} [b] ($s^{-1}site^{-1}$)	2.5 ± 0.3	0.59	0.71 ± 0.12	0.38 ± 0.04	0.68 ± 0.12	1.14 ± 0.15	0.88 ± 0.12	0.47 ± 0.08	0.36 ± 0.05	0.36 ± 0.22
$K_{m,ATP}$ (μM)	9.5 ± 0.4 [b]	31	13 ± 2	29 ± 16	11 ± 2	29 ± 4.5	25 ± 3	16 ± 2	29 ± 25	45 ± 4
$K_{0.5,MT}$ (μM)	0.7 ± 0.6 [b]	0.004	0.33 ± 0.31	0.08 ± 0.03	1.8 ± 0.3	0.07 ± 0.04	0.05 ± 0.04	0.08 ± 0.04	1.7 ± 0.5	1.3 ± 0.9

[a] Eg5 constants were previously determined at 68 mM ionic strength (21-23).
[b] Average fit constants ± standard deviation of the mean. *n* = 3–6 experimental data sets each condition.
